# Th17-inducing autologous dendritic cell vaccination promotes antigen-specific cellular and humoral immunity in ovarian cancer patients

**DOI:** 10.1038/s41467-020-18962-z

**Published:** 2020-10-14

**Authors:** Matthew S. Block, Allan B. Dietz, Michael P. Gustafson, Kimberly R. Kalli, Courtney L. Erskine, Bahaaeldin Youssef, Geraldine V. Vijay, Jacob B. Allred, Kevin D. Pavelko, Michael A. Strausbauch, Yi Lin, Megan E. Grudem, Aminah Jatoi, Carolyn M. Klampe, Andrea E. Wahner-Hendrickson, S. John Weroha, Gretchen E. Glaser, Amanika Kumar, Carrie L. Langstraat, Mary L. Solseth, Michael C. Deeds, Keith L. Knutson, Martin J. Cannon

**Affiliations:** 1 Department of Oncology, Mayo Clinic, Rochester, MN 55905 USA; 2 Department of Laboratory Medicine and Pathology, Mayo Clinic, Rochester, MN 55905 USA; 3 Department of Immunology, Mayo Clinic, Rochester, MN 55905 USA; 4 Department of Immunology, Mayo Clinic, Jacksonville, FL 32224 USA; 5 Mayo Clinic Cancer Statistics, Mayo Clinic, Rochester, MN 55905 USA; 6 Immune Monitoring Core, Mayo Clinic, Rochester, MN 55905 USA; 7 Department of Medicine, Mayo Clinic, Rochester, MN 55905 USA; 8 Department of Obstetrics and Gynecology, Rochester, MN 55905 USA; 9 Department of Microbiology and Immunology, University of Arkansas for Medical Sciences, Little Rock, AR 72205 USA

**Keywords:** Ovarian cancer, Cell vaccines

## Abstract

In ovarian cancer (OC), IL-17-producing T cells (Th17s) predict improved survival, whereas regulatory T cells predict poorer survival. We previously developed a vaccine whereby patient-derived dendritic cells (DCs) are programmed to induce Th17 responses to the OC antigen folate receptor alpha (FRα). Here we report the results of a single-arm open-label phase I clinical trial designed to determine vaccine safety and tolerability (primary outcomes) and recurrence-free survival (secondary outcome). Immunogenicity is also evaluated. Recruitment is complete with a total of 19 Stage IIIC-IV OC patients in first remission after conventional therapy. DCs are generated using our Th17-inducing protocol and are pulsed with HLA class II epitopes from FRα. Mature antigen-loaded DCs are injected intradermally. All patients have completed study-related interventions. No grade 3 or higher adverse events are seen. Vaccination results in the development of Th1, Th17, and antibody responses to FRα in the majority of patients. Th1 and antibody responses are associated with prolonged recurrence-free survival. Antibody-dependent cell-mediated cytotoxic activity against FRα is also associated with prolonged RFS. Of 18 patients evaluable for efficacy, 39% (7/18) remain recurrence-free at the time of data censoring, with a median follow-up of 49.2 months. Thus, vaccination with Th17-inducing FRα-loaded DCs is safe, induces antigen-specific immunity, and is associated with prolonged remission.

## Abstract (web summary)

The folate receptor alpha (FRα) is overexpressed in the majority of high-grade serous ovarian cancers and has been proposed as a candidate vaccine antigen. Here the authors report the safety and immunogenicity of Th17-inducing dendritic cells pulsed with FRα-derived epitopes in an early phase I clinical trial with ovarian cancer patients.

## Introduction

Because ovarian cancer (OC) is most frequently detected only after peritoneal dissemination, it is the most lethal gynecologic malignancy, with a 5-year survival rate of 47.8%^1^. The majority of patients with advanced OC achieve a complete remission from cancer after primary or interval cytoreductive surgery and chemotherapy with a platinum-taxane doublet. However, most patients develop recurrence, with a median time from diagnosis to recurrence of 10.3 months in patients who are candidates for primary debulking surgery^2^. There is thus an urgent need for new therapies that complement surgery and chemotherapy to reduce the recurrence rate of OC.

The magnitude and nature of the host immune response to OC is widely recognized to be associated with clinical outcomes. Zhang and colleagues first demonstrated that T cell infiltration is positively associated with OC survival, which was subsequently confirmed in other studies^3–6^. Given the strong association between TILs and survival in OC, several groups have tested therapeutic vaccines in OC patients, with the goal of improving clinical outcomes by expanding OC-specific T cells. We previously identified the folate receptor alpha (FRα) as a candidate vaccine antigen, as FRα is overexpressed in the majority of high-grade serous OC patients, but is otherwise very restricted in normal tissues^7^. Furthermore, OC patients with high FRα expression have reduced relapse-free survival (RFS) and overall survival (OS) rates relative to patients with low FRα expression^8^. Numerous other studies have validated FRα as a target antigen for diverse approaches to OC immunotherapy, including tumor vaccines, antibody treatment, and CAR-T cells^9–19^. We tested OC (and breast cancer) patients for spontaneous immune responses to putative HLA class II-binding epitopes and identified five FRα-derived epitopes to which patients (but not healthy volunteers) exhibit an immune response^20^. We focused on HLA-II class epitopes, given the central role of CD4 T cells in immune surveillance and coordination of effective immunity against infectious diseases and cancer^21–24^. We tested the five FRα-derived epitopes as a vaccine in combination with granulocyte-macrophage colony-stimulating factor (GM-CSF) in patients in remission from OC or breast cancer (NCT01606241)^25^. The vaccine was well tolerated, with no treatment-related severe systemic toxicities, and over 90% of patients demonstrated new or augmented immunity to FRα, as measured by interferon gamma (IFN-*γ*) ELIspot testing^25^. While the study was not powered to assess clinical outcomes, the median RFS among 10 patients with OC in the first remission was 528 days^25^.

Although OC-reactive TILs are generally associated with favorable outcomes, tumor-infiltrating regulatory T cells (Tregs), which inhibit effector T cell function via recruitment of myeloid-derived suppressor cells, are linked to poor survival in OC^26–28^. In contrast, high interleukin 17 (IL-17) concentrations in ascites of OC patients are associated with prolonged OS^29–31^. Furthermore, the frequency of infiltrating T lymphocytes capable of secreting IL-17, known as Th17 cells, has been inversely related to Treg frequencies in OC, suggesting that Th17 cell expansion might counteract Treg-mediated suppression of OC-targeted T cell responses^29^. Collectively, these observations suggest that tumor vaccine strategies designed to preferentially activate and expand OC antigen-specific Th17 responses may afford the prospect of therapeutic benefit.

Dendritic cell (DC) vaccination has been tested for the treatment of many other malignancies, but clinical studies of DC vaccination in OC have been limited. Prior studies have shown that DC vaccinations in OC patients are generally well-tolerated and immunogenic in at least a proportion of patients, while also providing evidence of epitope spreading^32–35^. In the most comprehensive study reported to date, the detection of tumor antigen-specific T cell responses following DC vaccination correlated with significantly improved clinical outcome, notably for a cohort of patients who received DC vaccination combined with bevacizumab and cyclophosphamide^36^. While encouraging, these DC vaccine studies were not designed to induce Th17 immune responses, and no subjects were tested for tumor antigen-specific Th17 immunity. We have shown that pharmacologic inhibition of the mitogen-activated protein kinase (MAPK) family member p38 in ex vivo generated DCs enables them to promote a Th17-enriched immune response. In brief, treatment of monocyte-derived DCs with a p38 inhibitor in combination with IL-15 diminished PD-L1 expression and indoleamine 2,3-dioxygenase function, and favored stimulation of ovarian tumor antigen-specific Th1/Th17 CD4^+^ T cell responses, while also reducing CD4^+^Foxp3^+^ Treg expansion^37^. Based on the promising clinical immunogenicity of our five-peptide FRα vaccine, as well as the encouraging preclinical studies on Th17-inducing DC stimulation of ovarian tumor antigen-specific CD4^+^ T cell responses, we conducted a pilot clinical trial testing a vaccine composed of monocyte-derived autologous Th17-inducing DCs pulsed with FRα epitopes. Rather than conducting the trial in OC patients with the measurable disease following recurrence, we elected to take advantage of a window of opportunity when patients have minimal residual disease following first-line surgery and chemotherapy. These patients are more likely to be immunocompetent and responsive to DC vaccination, and less likely to suffer from comorbidities associated with disease burden, yet they retain a very high likelihood of disease recurrence and progression within 12–18 months. The objectives of the present study are to evaluate the safety and immunogenicity of Th17-inducing DC vaccination in patients in remission after completion of primary or interval debulking surgery and initial chemotherapy for FIGO stage IIIC or IV OC. A secondary goal is to document recurrence-free survival. We report that Th17-inducing DC vaccination is well tolerated and induces robust and durable IFN-*γ*^+^ and IL-17^+^ T cell responses to FRα in most patients, and also induces FRα-specific antibody responses that are associated with extended RFS.

## Results

### Patient characteristics

Nineteen patients with stage IIIC-IV ovarian, fallopian tube, or primary peritoneal cancer who had completed standard surgery and first-line chemotherapy were enrolled. Baseline characteristics including age, histotype, and the primary site of disease, the timing of debulking surgery (primary debulking versus neoadjuvant chemotherapy), debulking status, and number of cycles of chemotherapy are listed in Table 1.

**Table 1:** Baseline patient characteristics.

Median age (range)	58 (45–77)
Stage	
IIIC	16
IV	3
Histotype	
High-grade serous	18
Mixed	1
Primary	
Ovary	16
Fallopian tube	2
Peritoneum	1
Neoadjuvant chemotherapy	
No	14
Yes	5
Debulking status	
Primary, R0	9
Primary, <1 cm	4
Primary, >1 cm	1
Interval, R0	5
Median cycles of chemotherapy (range)	6 (6–9)

### DC vaccination is feasible and safe

Patients were treated with five cycles of induction vaccination, given every 3 weeks (1–2 × 10^7^ DCs/dose, delivered intradermally). Patients who completed the induction phase of DC vaccination without confirmation of recurrence underwent repeat disease evaluation and maintenance DC vaccination every three months for up to two years from study enrollment. Of 19 patients enrolled, adequate manufacturing of vaccine, with release criteria met for at least 12 aliquots of FRα-loaded DCs, was achieved after a single apheresis in 18 patients. One patient’s PBMCs were contaminated after the first apheresis collection. A second collection yielded only enough DCs for five aliquots of FRα-loaded DCs. As such, this patient underwent 5 cycles of vaccine priming, and then was subsequently observed and did not receive maintenance vaccine treatment. Vaccine manufacturing results are shown in Supplementary Table 1.

All 19 patients were evaluable for safety. A summary of adverse events, as graded via CTCAE v 4.0, is listed in Supplementary Table 2. No dose-limiting toxicity (DLT), and no Grade 3 or higher adverse events were observed. Grade 1 injection site reactions were observed in 94.7% of patients. Other than injection site reactions, the most common adverse events seen were arthralgias (52.6% Grade 1, 15.8% Grade 2), pruritus (21.1% Grade 1, 5.3% Grade 2), and myalgias (26.3% Grade 1). All adverse events were transient in nature.

Subsequent to enrollment, one patient was deemed ineligible for participation and was therefore not evaluable for clinical or immunologic outcomes. The patient completed the induction phase immunizations but was retrospectively found to be ineligible due to a history of autoimmune disease. This patient did not experience any Grade 2 or higher adverse events.

### Immunization generates IFN-*γ*^+^ and IL-17^+^ T cell responses

**Fig. 1:**
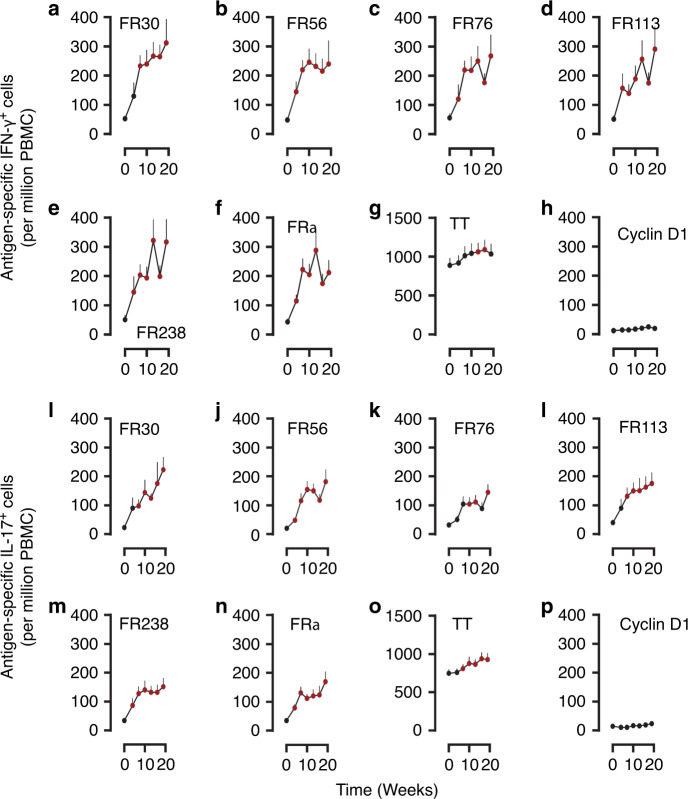
Immunization with vaccine generates both IFN-γ^+^ and IL-17^+^ T cell responses. **a**–**h** Induction time courses of antigen-specific IFN-*γ*^+^ T cell frequencies (T cells per million PBMCs, mean + s.e.m.) for FRα epitopes FR30, FR56, FR76, FR113, FR238 FRα protein, TT, and control cyclin D1 peptide, respectively, in 18 evaluable patients. **i**–**p** Induction phase time courses of antigen-specific IL-17^+^ T cell frequencies (T cells per million PBMCs, mean + s.e.m.) for the same antigens in the same patients. The first time point is prior to apheresis; the second following apheresis and prior to Cycle 1 of vaccine treatment, and the remainder following Cycles 1–5 of vaccine induction. Reddened symbols indicate *p* < 0.008 (Bonferroni corrected) significance by Wilcoxon matched pairs two-sided test, compared to the baseline value for that antigen. Exact *P* values are indicated in Supplementary Tables 3 and 4.

Data from the ineligible patient were excluded from immune response and survival analyses. As such, 18 patients were evaluated for RFS and for immune responses to the vaccine. Patients were evaluated for T cell responses to each of the FRα vaccine peptides and to recombinant FRα protein prior to immunization, during and after completion of the induction phase of the DC vaccine schedule (i.e., the first 5 DC vaccinations). ELIspot assays showed that the frequency of both IFN-*γ*^+^ (Fig. 1a–f and Supplementary Table 3) and IL-17^+^ (Fig. 1i–n and Supplementary Table 4) responses to each of the FRα epitopes and the FRα protein increased throughout vaccine induction. In addition, the mean frequency of antigen-specific T cells increased modestly following apheresis (before vaccine treatment), a finding consistent with prior analyses, but increased more sharply after a single vaccine treatment in most cases^38^. While minor increases in the frequencies of tetanus toxoid (TT)-recognizing T cells (Fig. 1g, o and Supplementary Tables 3 and 4) were seen (patients received booster injections of tetanus toxoid at study entry if not given in the prior 2 years), responses to Cyclin D1 did not increase during vaccine induction (Fig. 1h, p and Supplementary Tables 3 and 4).

### DC vaccination induces T cell responses in most patients

**Fig. 2:**
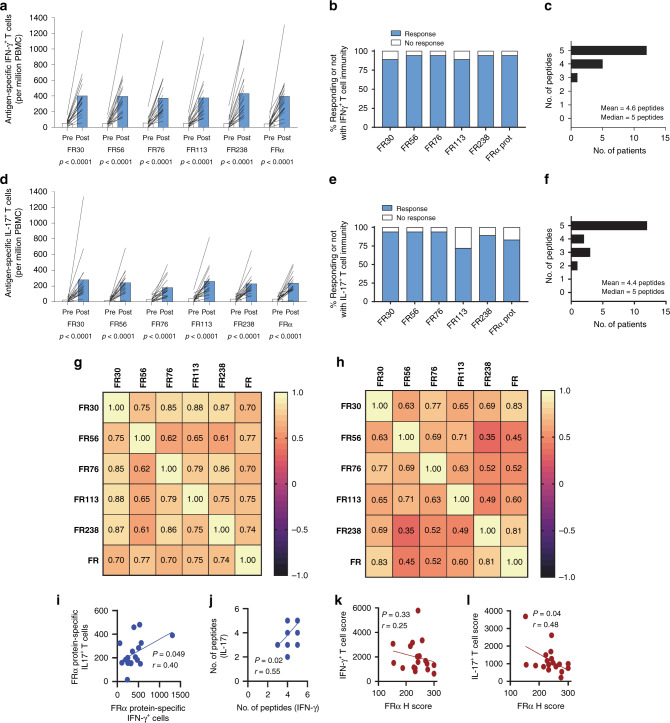
T cell immunity is generated in the vast majority of patients. **a**, **d** The mean (*n* = 18) pre-immunization (Pre) and highest post-vaccination (Post) frequency of antigen-specific IFN-*γ*^+^ (**a**) or IL-17^+^ T (**d**) cell frequencies (per million PBMCs plated) that recognize the vaccine epitopes as well as the whole FRα protein. Post-vaccination samples included up to week 19. P values were calculated using the 2-sided test Wilcoxon matched pairs at a significance level of *P* ≤ 0.05. **b**, **e** The % of patients that responded to vaccine epitopes with IFN-*γ*^+^ or IL-17^+^ T cell responses, respectively. **c**, **f** The distribution of IFN-*γ*^+^ and IL-17^+^ T cell responses to the individual epitopes, respectively. **g** Correlation analysis heatmap comparing the magnitude of maximal peptide-specific IFN-*γ*^+^ T cells to each other and to the maximal FRα protein-specific IFN-*γ*^+^ T cell response. Inset values are Spearman’s Rho. **h** The same for IL-17^+^T cell responses. All correlations ≥0.45 were *P* < 0.05 (Benjamini-Hochberg adjusted two-sided test, exact *P* values are indicated in Supplementary Tables 5 and 6). **i** Correlation plot between the protein-specific IFN-*γ*^+^ T cell response and the IL-17 analyses between the highest FRα protein-specific IFN-*γ*^+^ and highest FRα protein-specific IL-17^+^ T cells. **j** Correlation analysis between the number of peptides to which IFN-*γ*^+^ T cells were generated and the number of peptides to which IL-17^+^ T cells were generated. **k** Correlation plots between the vaccine IFN-*γ* score (The sum of the individual patient T cell response to the epitopes) and tumor FRα expression. Inset values for (**i**–**k**) are Spearman’s Rho coefficient (*r*) and *P* value. **l** Correlation plots between the vaccine Th17 score (The sum of the individual patient T cell response to the epitopes) and tumor FRα expression. Inset values are Pearson’s Rho coefficient (r) and *P* value. Each symbol in (**i**–**l**) represents a unique patient (*n* = 18 for each panel). In some cases the symbols overlap obscuring each other. Inset best-fit lines were calculated with non-linear least squares regression and intended for data trend visualization.

Comparisons of pre- and high post-vaccine T cell frequencies showed significant increases in frequencies of IFN-*γ*^+^ T cells specific for each FRα peptide and for FRα protein (Fig. 2a), with 89% of patients (16/18) exhibiting IFN-*γ*^+^ T cell responses to FRα protein (defined as at least a tripling of antigen-specific T cells) during at least one-time point during the induction phase of vaccination (Fig. 2b). IFN-*γ*^+^ T cell response rates to the five FRα epitopes used in the vaccine ranged between 89–100% and the median number of epitopes to which patients responded was 5 (Fig. 2c). Similarly, IL-17^+^ T cell immune responses to FRα protein were elevated in 78% of patients (14/18), with responses to all of the individual epitopes seen in 72–94% of patients (Fig. 2d–f).

Correlation analysis was used to determine which of the epitopes are critical to the generation of immunity to whole FRα protein. As shown in Fig. 2g–h and Supplementary Fig. 1 and Supplementary Tables 5–6), the frequencies of epitope-specific T cells generally correlated tightly with the frequency of FRα protein-specific T cells, suggesting that all were relevant to generating immunity to the natural antigen. Despite that, the correlations of the IL-17^+^ T cell response were more modest than the IFN-*γ* T cell responses, possibly suggesting that the IL-17^+^ T cell responses were of lower avidity. Furthermore, there were moderate to strong correlations between the responses to the individual epitopes emphasizing the degenerate nature of the epitope pool. Thus, the patients that responded well to one of the epitopes responded well to the others. The magnitude and frequency of IL-17^+^ T cell responses appeared highly correlated with IFN-*γ* responses (Fig. 2i–j). Although variable, FRα expression was observed on all patient tumor specimens. High FRα expression levels in the primary tumor affected the induction of IL-17^+^ but not IFN-*γ*^+^ T cells (Fig. 2k–l).

### Immunization generates cognate antibody immunity

**Fig. 3:**
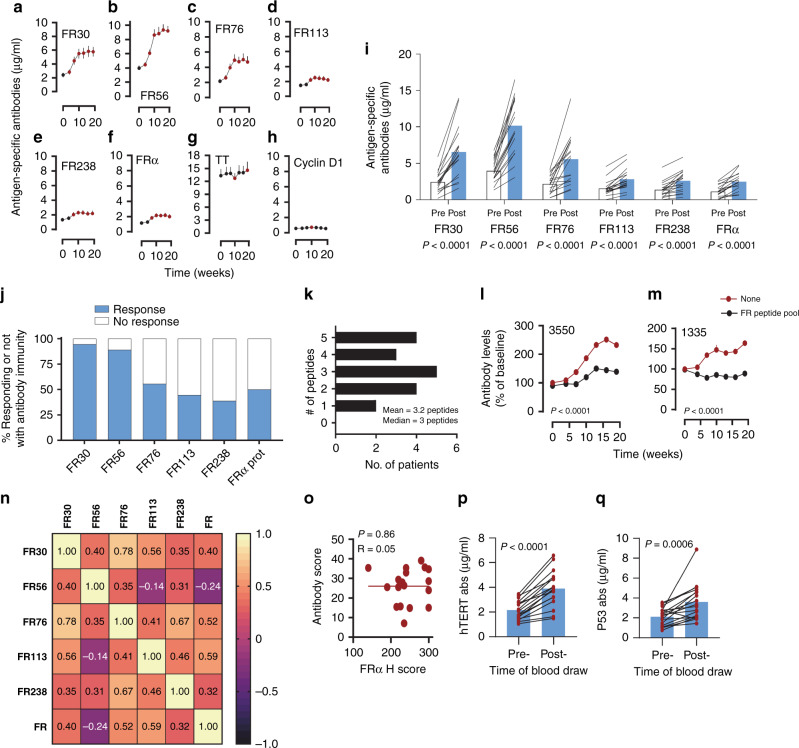
DC vaccines induce cognate antibody immunity. **a**–**h** Induction phase times courses of antigen-specific antibody levels (μg/ml, mean + s.e.m.) for FRα epitopes FR30, FR56, FR76, FR113, FR238 and FRα protein, TT, and control cyclin D1 peptide, respectively, in 18 evaluable patients. Reddened symbols indicate *P* < 0.008 (Bonferroni corrected) significance by Wilcoxon matched pairs two-sided test, compared to the baseline value for that antigen. Exact *P* values are indicated in Supplementary Table 7. **i** The mean (*n* = 18) pre-immunization (Pre) and highest post-vaccination (Post) frequency of antigen-specific antibody levels (μg/ml) to the vaccine epitopes as well as the whole FRα protein. Post-vaccination samples included up to week 19. *P* values were calculated using the two-sided test Wilcoxon matched pairs at a significance level of *P* ≤ 0.05. **j** % of patients that responded to vaccine epitopes with antibody responses at the ≥2-fold increase threshold. **k** The distribution of antibody responses to the individual epitopes. **l**, **m** Levels of FRα protein-specific antibodies over the time course of the 19-week vaccine period, expressed a % of baseline, for two patients. Plasma was left either untreated (red line) or pre-absorbed (black line) with pooled vaccine peptides prior to ELISA. Each data symbol represents mean (+s.e.m.) of two replicates. *P* values were calculated using two-sided two-way analysis of variance. **n** Correlation heatmap comparing the magnitude of maximal peptide-specific antibody levels to the maximal FRα protein-specific and epitope-specific antibody levels. Inset values are Spearman’s Rho. Correlations >0.56 were *P* < 0.05 (Benjamini-Hochberg two-sided adjusted *P* values). Exact *P* values are indicated in Supplementary Table 8. **o** Correlation plot between the vaccine antibody score (sum of the individual patients response to each epitope) and tumor FRα expression. Inset values are Pearsons’s Rho coefficient and *P* value. Each symbol represents a unique patient and the inset line is best-fit lines was calculated with non-linear least squares regression and intended for data trend visualization. **p**, **q** Pre- and post-immunization (19-week time point) serum levels of IgG antibodies specific for p53 and hTERT, respectively, in each of the 18 patients. Inset blue bar represents the mean levels of antibodies for all patients at pre- and post-immunization. *P* values comparing the means were calculated with a two-sided paired Student’s *t* test.

FRα-specific antibody responses to FRα epitopes and protein were also induced during vaccination and generally peaked by the time of the third immunization (Fig. 3a–f and Supplementary Table 7). Antibody responses to TT were slightly elevated at 19 weeks, likely due to immunization, whereas antibodies to control cyclin D1 peptide were low and did not boost as a result of immunization (Fig. 3g–h and Supplementary Table 7). The majority of patients demonstrated elevated antibody immunity to all of the peptides at least one-time point during immunization, and antibody responses targeting native FRα protein (defined as a doubling compared with baseline) were observed in 50% of patients, with antibody responses to individual vaccine epitopes seen in 39–94% of patients (Fig. 3i–j). The stronger responses to FR30 and FR56 may in part be attributed to higher baseline antibody titers pre-vaccination (Fig. 3i), suggesting a higher level of natural immunity to epitopes within these peptides. The median number of epitopes eliciting a response in a given patient was 3, with a range of 1–5 epitopes inducing a response (Fig. 3k). For two patients, we assessed the change in FRα protein-specific antibody concentration over time after removing those antibodies capable of binding the vaccine epitopes (Fig. 3l–m). These data demonstrate that while the total antibody to FRα protein increased during vaccine induction, the frequency of antibodies that recognized the protein but not the vaccine peptides did not increase. As such, we conclude that the increase in protein-specific antibodies was due to increases in vaccine peptide-specific antibodies. The post-vaccination levels of FR76- and FR113-specific antibodies were correlated (*P* < 0.05 unadjusted for multiple testing) with increasing levels of protein-specific antibodies suggesting that these epitopes are biologically relevant in vivo (Fig. 3n, Supplementary Fig. 1K–O and Supplementary Table 8). Unlike IL-17^+^ T cell immunity, the generation of antibodies by the vaccine was not impacted by levels of tumor expression of the FRα. The generation of antibodies was not limited to FRα. Specifically, we observed epitope spreading to other previously identified OC-associated tumor antigens, hTERT, and p53 (Fig. 3p–q)^39,40^.

### Immunization appears to protect against recurrence

**Fig. 4:**
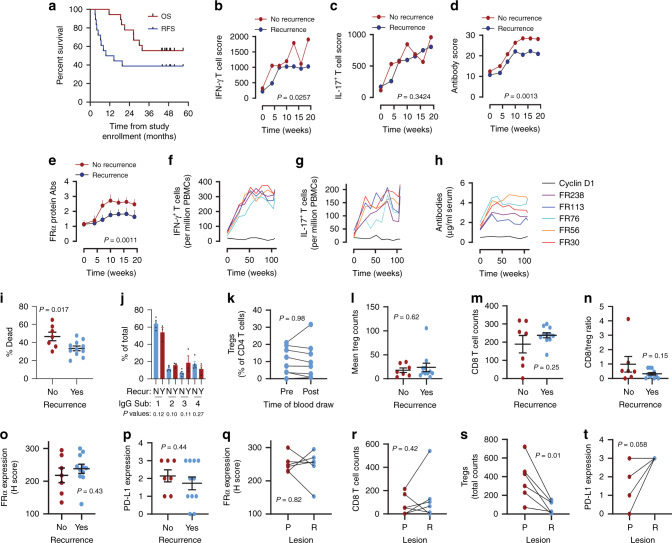
Patients who develop persistent broad immunity against FR*α* appear to be protected against recurrence. **a** The RFS and the OS from the time of study enrollment (4–20 weeks after first-line chemotherapy) for all patients eligible for efficacy analysis (*n* = 18) via Kaplan–Meier analysis. **b**–**d** Compare IFN-*γ*, IL-17, and antibody immune response scores (sum of the mean immune responses to the epitopes), respectively, of those who recurred (blue symbols, *n* = 11) and those who did not recur (red symbols, *n* = 78). **e** Compares the mean (+s.e.m.) antibody levels to whole FRα protein in those that recurred and those that did not recur. *P* values for (**b**–**e**), comparing the separation of the two curves, are calculated using a two-way ANOVA test. Expanded datasets are shown in Supplementary Fig. 2. **f**–**h** Maintenance phase time courses of antigen-specific IFN-*γ*^+^ and IL-17^+^ T cell frequencies (T cells per million PBMCs) or the antibody concentrations for FRα epitopes FR30, FR56, FR76, FR113, and FR238, and control cyclin D1 peptide, respectively, in eight evaluable patients. **i** The individual and mean (±s.e.m.) percentage of dead cells killed through ADCC assayed with post-immunization (Week 19) plasma samples from patients who did not recur (*n* = 7) and did recur (*n* = 11). **j** Bars (mean + s.e.m.) show relative levels of IgG subtypes (IgG1-4) in *n* = 5 patients with recurrence and *n* = 3 patients with no recurrence. **k** The pre- and post-vaccination (i.e., week 19) circulating levels of Tregs in eight evaluable patients. **l**–**p** The individual and mean (±s.e.m.) tumor Treg counts, tumor CD8 T cell counts, CD8/Treg ratio, tumor FRα, and PD-L1 expression, respectively, in the primary tumors of patients who did (Yes) and did not (No) have recurrence. **q**–**t** Compare the tumor FRα expression, CD8 T cell infiltration, Treg infiltration, and PD-L1 expression, respectively in primary and corresponding recurrent tumors from *n* = 5–6 patients with recurrence. *P* values for (**i**–**t**) were calculated using the two-sided paired Student’s *t* test. Symbols in some cases overlap obscuring the number of patients.

RFS and OS are shown in Fig. 4a. The median RFS was 12.1 months, while the median OS was not reached. At the time of data cut-off, 38.9% of at-risk patients remained alive and free from recurrence, and no patient who did not recur during the vaccine maintenance period has recurred at a later time (median follow-up: 49.2 months). While there was no comparator arm in the present trial, RFS compared favorably to that observed (≤15% progression-free survival at 36 months following randomization) in the GOG-0218 bevacizumab phase III clinical trial^2^. Neither the dose of DCs nor the delivery method (needle vs. hMTS, see “Methods”) was associated with recurrence.

The cumulative (i.e., sum of the mean responses to vaccine epitopes) IFN-*γ*^+^ T cell and antibody responses to FRα vaccine epitopes were significantly elevated in those patients who did not experience recurrence relative to those with recurrence, but not for IL-17+ T cell responses (Fig. 4b–d, Supplementary Fig. 2A–S). The levels of antibodies to the whole FRα protein were also significantly (*p* = 0.0011) higher in patients who did not recur (Fig. 4e).

Levels of FRα-specific T cells and antibodies remained increased relative to baseline throughout the maintenance period of study participation (weeks 27–104) (Fig. 4f–h, Supplementary Fig. 3A–F, I–N, Q–V, and Supplementary Tables 9–11). By contrast, levels of T cells and antibodies specific for Cyclin D1 (negative control peptide) and TT (positive control) remained relatively stable during this period (Supplementary Fig. 3G–H, O–P, W–X and Supplementary Tables 9–11).

Analysis of antibody function using ADCC assays revealed that patients without recurrence demonstrated higher levels of ADCC activity against FRα-expressing target cells relative to those that recurred (*p* = 0.017) (Fig. 4i). This increased ADCC activity did not appear, however, to be attributable to a differential distribution of the FRα-specific antibodies among different subtypes of IgG (Fig. 4j).

A reduction in circulating Tregs following vaccination did not correlate with RFS. While previous studies have shown an inverse relationship between IL-17^+^ T cell immunity and levels of Tregs, no statistically significant decline in Tregs was observed, although slight declines were observed in most patients (Fig. 4k, i). Pretreatment levels of intratumoral levels of CD4 Tregs or CD8 T cells as well as the CD8/Treg ratios, all parameters are previously shown as prognostic, were also not associated with whether an individual recurred (Fig. 4l–n)^26–28^. Similarly, pretreatment levels of intratumoral FRα and PD-L1 expression were also not associated with recurrence (Fig. 4o–p)^4^. The lack of association of these previously identified prognostic parameters may reflect the wide variation observed with these markers and the limited sample size of the present trial.

Analysis of recurrent tumors in a subset of patients whose tissue was available showed that loss of FRα expression or CD8 T cell infiltration did not associate with recurrent disease (Fig. 4q, r). However, Tregs were significantly reduced in recurrent lesions in patients that progressed (Fig. 4s). Lastly, PD-L1 was increased in three of five evaluable recurrent tumors relative to baseline tumor levels (Fig. 4t). Any immunological advantage from loss of Tregs may be offset by the gain of PD-L1 expression and the possibility that recurrent tumors were immunoedited as a result of vaccination, potentially resulting in disease relapse.

## Discussion

Over the past two decades there have been significant advances in the understanding of T cell biology. The older Th1/Th2 dichotomy paradigm has been largely displaced in favor of the idea that there are several distinct subsets of T cells (e.g., Th22, Th17, Th9). Each T cell subset may indeed have distinct roles in different tumor microenvironments. These advances in T cell biology present new opportunities for the development of vaccines that favor the induction of biologically relevant T cell subsets that yield clinically meaningful results. Evidence for beneficial or detrimental roles for Th17 responses in cancer is often idiosyncratic and contradictory^2^. Th17 T cell associated inflammation may be pathologic in some tumor phenotypes^41,42^, but correlates with favorable outcomes in OC. Kryczek and colleagues have noted that Th17-derived IL-17 and IFN*γ* induce the production of CXCL9 and CXCL10, which promote effector cell migration into ovarian tumors^29^. Furthermore, Th17 cells retain antitumor efficacy and resist senescence more effectively than Th1 cells after long-term expansion^43^, and human Th17 cells may be long-lived effector memory cells^44^. Consistent with this philosophy, we formulated a Th17-inducing vaccine specifically for OC to prevent disease recurrence based on prior observations that IL-17 and Th17 T cells are associated with improved survival^29–31^. The rationale for our approach was based in part on the development of a degenerate epitope pool derived from FRα, and in part on our prior DC studies that identified actionable targets relevant to Th17 generation^7,25,37^. In contrast to prior use of vaccines in the bulky disease setting, the clinical setting was based on observations that vaccines work optimally in the disease prevention setting, thus we selected patients with stage IIIC/IV OC with no radiologic evidence of disease and normal CA125 values. This disease setting is advantageous because there is a reasonable prospect of immune competence with reduced disease-related comorbidities and little or no tumor-induced immune suppression.

We found that Th17-inducing DC vaccination targeting the FRα was well tolerated, with only transient grade 1 and 2 toxicities, mostly related to injection site reactions. DC vaccine rapidly and efficiently induced FRα-specific IFN-*γ*^+^ and IL-17^+^ T cell responses to the majority of FRα epitopes and FRα protein in most patients, which is consistent with the degenerate nature of the epitope pool employed^25^. Perhaps one of the more important findings in the present study was the observed 39% recurrence-free survival rate (calculated from the time of study enrollment rather than from the time of diagnosis) over a period exceeding 48 months. We consider the rate of prolonged OC remission to be highly encouraging, relative to historical controls^2,45,46^. We do, however, recognize the limitations of stochastic variation with a small sample size, and we cannot exclude the possibility of patient selection bias. Nonetheless, there is compelling reason to believe that the improved RFS resulted from vaccination given the association with the development of durable FRα-specific IFN-*γ*^+^ T cell, antibody immunity, and higher ADCC responses, but not the previously identified prognostic immune biomarkers (i.e., CD8 T cell infiltration, Treg infiltration)^5,20,25,27,28^. It is not clear why the induction of FRα-specific IL-17^+^ T cell responses were not correlated with RFS. One possible explanation is that the high individual variation in response amplitude at different time-points, coupled with the small sample size, contributed to the lack of significance. Another possibility that IL-17^+^ T cell responses may be necessary but not sufficient themselves to contribute to disease control.

A unique finding was that Th17-inducing DC vaccination-stimulated FRα-specific antibody responses against the constituent FRα epitopes as well as the protein. This appears to be related to the mode of vaccination, considering that no antibodies were generated when the epitopes were delivered as free peptides along with GM-CSF as adjuvant^25^. We also observed some evidence of epitope spreading, in that DC vaccination was associated with increased antibody titers to p53 and hTERT. However, these antibody responses were not associated with prolonged RFS, possibly because p53 and hTERT are intracellular antigens that are not accessible to antibodies. The observation that Th17-inducing DC vaccination-stimulated antibody responses is unique but not unexpected, given that DC vaccine stimulation of robust antibody responses has previously been described^47^. Th17 T cells can provide effective help for B cell proliferation, antibody production, and class switching, and thus it is probable that Th17-inducing DC induction of FRα-specific helper T cell responses could promote antibody production^48^. There is also prior evidence that DCs can act more directly to promote B cell responses. Earlier studies have shown that DCs can retain unprocessed antigens for stimulation of B cells, along with signals that promote isotype switching, but that T cell help is required for antibody production^49^. DCs possess non-degradative antigen uptake pathways that allow the direct presentation of antigen to B cells^50^. Although the prevailing mechanisms behind DC stimulation of FRα-specific antibody production in this clinical trial are unknown, cooperative T-dependent and T-independent pathways of DC stimulation may contribute to the antibody response.

The ELIspot analysis was done on whole, unfractionated PBMC (i.e., including both CD4 and CD8 T cells). It is possible that Th17-inducing DC vaccine could have elicited CD8 T cells, in addition to CD4 T cells, providing an opportunity for future studies. Both Th1 and Th17 helper T cells are known to induce cytotoxic CD8 T cell responses which may have participated in an anti-tumor immune response^51–53^. Indeed, we have previously shown that HLA class II epitopes can contain embedded HLA class I epitopes^54^. Additional opportunities also include the role of other T cell phenotypes that are important in tumor eradication, including Th2, Th9, Tfh, and Th22^55^.

Our results both support and contrast with the findings from another recently reported study, which found that DC vaccination combined with bevacizumab alone or with bevacizumab plus low dose cyclophosphamide in recurrent OC patients was well tolerated and stimulated T cell immunity against vaccine antigen (an oxidized tumor cell lysate) and autologous tumor cells^36^. These authors found that the highest frequency of immune responses was seen in patients that received DC vaccination combined with cyclophosphamide, and that ex vivo T cell responses to tumor antigen-loaded DCs or autologous tumor cells were associated with significantly better recurrence-free survival, recorded for up to 24 months. Notably, those patients that received the triple combination treatment of DC vaccination, bevacizumab, and cyclophosphamide also demonstrated improved OS over 24 months compared with those that only received DC vaccine vaccination plus bevacizumab. In comparison, we observed markedly higher immune response rates from DC vaccination alone (89% for IFN*γ*-producing T cell responses and 78% for IL-17-producing T cell responses, respectively, to FRα protein), and we also report an overall RFS rate of 39% over a period exceeding 49 months for 18 evaluable patients. However, comparisons between response rates for the two trials are difficult to interpret, given the differences in patient populations. Whereas Tanyi and colleagues treated patients with measurable recurrent OC^36^, we based eligibility on patients with no radiologic evidence of disease and with normal baseline CA125 levels following first-line surgery and chemotherapy. Consequently, our patient cohort likely enjoyed better health and immune competence, with reduced comorbidities that may have limited responsiveness to DC vaccination, hence the more favorable outcomes.

A potentially important finding from our study is the observation that levels of FRα-specific Th17 T cell immunity generated by vaccination were related to the levels of FRα expression in the tumor. Specifically, significantly lower IL17^+^ T cell immunity was observed in those patients that demonstrated higher levels of FRα expression in the primary tumor. A similar trend was observed for IFN*γ*^+^ T cell immunity, but this did not reach statistical significance. In contrast, FRα-specific antibody responses did not show any association with tumor FRα expression levels. Because the tumors had been resected for several months at the time of entry onto the study, it does not seem likely that the tumor tissue had a direct effect on the generation of immunity. Rather it is possible that the T cell compartment (but not the B cell compartment and humoral immunity) was preferentially preconditioned or tolerized by expression of the FRα during tumor growth. Considering that the inverse relationship between T cell response and antigen expression was observed only in the Th17 compartment, one possibility is a role for the T cell interconversion from Tregs to Th17 T cells. Although further validation is required to demonstrate interconvertibility, support for this hypothesis comes from the longstanding knowledge of the plasticity of Th17 and Treg responses^56^. Komatsu, for example, demonstrated antigen-specific Tregs can be converted to pathogenic autoreactive Th17 T cells, through modulation of FoxP3 stability, leading to increased autoimmune pathogenesis^57^. Thus, one possible explanation is that high-level FRα exposure may stabilize FoxP3 expression in FRα-specific Tregs preventing interconversion to Th17 T cells.

In the interim since this study commenced in 2014, others have investigated the use of PARP inhibitors and bevacizumab in the same population of patients (advanced OC following surgery and first-line chemotherapy). Bevacizumab has demonstrated improvement in progression-free survival (PFS) when given concurrently and after first-line chemotherapy for advanced OC patients and is now approved for use in this setting^2,58^. For patients with somatic or germline BRCA1 or BRCA2 mutations, olaparib has been shown to dramatically prolong progression-free survival (PFS)^46^. Olaparib has been approved as maintenance therapy for patients with a BRCA1 or BRCA2 mutation. In addition, niraparib has demonstrated prolongation of PFS in advanced OC patients unselected for BRCA1/BRCA2 mutation status and has been approved as post-first-line maintenance therapy^45^. While these advances are encouraging, no maintenance therapy to date has been demonstrated to provide a benefit in OS. As such, there is debate as to which patients should receive bevacizumab, a PARP inhibitor, both, or neither as maintenance therapy after surgery and first-line chemotherapy.

We recognize that the low patient enrollment and the potential for selection bias or stochastic variation in our pilot study limit the interpretation of immune and clinical correlates. Nonetheless, we conclude that Th17-inducing DC vaccination is well tolerated, highly immunogenic and is associated with clinical benefit in a subset of OC patients. The determination of candidate predictive biomarkers for clinical response to Th17-inducing DC vaccination will be an important future goal in our developing randomized Phase II clinical trial.

## Methods

### Patient selection and enrollment

This single-arm pilot study enrolled patients 18 years of age or older with histologically confirmed stage IIIC or stage IV epithelial ovarian, fallopian tube, or primary peritoneal cancer between 15 April 2014 and 22 June 2016. The full study protocol is available as Supplementary Note 1 in the Supplementary Information file. Patients were eligible if they completed primary or interval cytoreductive surgery and a course of 5–9 cycles of platinum-based chemotherapy (either neoadjuvant and adjuvant or all adjuvant) 4–20 weeks prior to study enrollment. Additional inclusion criteria consisted of having no evidence of disease by history, exam, CA-125, or CT scan; having an ECOG performance status of 0 or 1; having adequate hematologic, renal, and liver tests; anticipated survival >6 months; and willingness to undergo a tetanus booster vaccination if none had taken place within a year prior to study registration. Patients were excluded if they had serious comorbidities that would interfere with proper safety/toxicity assessments, if they were immunocompromised or on immunosuppressive therapy (including systemic steroids), or if they had a history of autoimmune disease. The protocol was approved by the Mayo Clinic Institutional Review Board, and assurances were filed with the Department of Health and Human Services; written informed consent to treatment and all study-related procedures was required prior to registration and study participation. All patients were seen and treated at the Mayo Clinic in Rochester, MN. The study was registered at clinicaltrials.gov as NCT02111941.

### Dendritic cell manufacturing

Study subjects underwent evaluation for the adequacy of veins for apheresis by a nurse with apheresis experience. Patients with veins deemed inadequate for apheresis were permitted to undergo placement of a temporary dialysis catheter for apheresis (removed the following day). Patients underwent collection of a 10-l volume of peripheral blood mononuclear cells (PBMCs). Collected PBMCs were transferred to the Mayo Clinic Immune Progenitor and Cell Therapeutics (IMPACT) Laboratory within 0.5 h of collection. DCs were manufactured according to protocols based on our previous work^37^. In brief, CD14^+^ monocytes were isolated via magnetic bead positive selection (CliniMACS Plus system, Miltenyi Biotec, Bergisch Gladbach, Germany) and were cultured with GM-CSF, interleukin 4 (IL-4), IL-15, and the p38 MAP kinase inhibitor methylsulanylimidazole (Calbiochem, San Diego, CA) for seven days. The culture medium was exchanged on days 3 and 5. On day 5, aliquots of cells underwent the addition of the maturation factors tumor necrosis factor alpha (TNFα), IL-1β, and prostaglandin E2 (PGE2); as well as loading with 1 of 5 FRα peptides (Supplementary Table 12). On day 7, epitope-loaded DC aliquots were pooled such that vaccine aliquots contained equal numbers of DCs loaded with each FRα epitope (total of 1–2 × 10^7^ cells/aliquot). Vaccine aliquots were frozen in CryoStor CS10 (BioLife Solutions, Bothell, WA). In order for the vaccine product to be released, cells had to meet purity and sterility assays including aerobic, anaerobic, endotoxin, and mycoplasma testing, as well as having at least 1–2 × 10^7^ cells with >70% purity (as measured by CD83 staining). If sufficient vaccine for 12 aliquots of FRα DCs did not meet release criteria, patients were allowed to undergo a second apheresis collection. Patients from whom at least 5 aliquots of FRα-loaded DCs were prepared were subsequently allowed to receive vaccine therapy.

### Vaccine treatment and clinical monitoring

Prior to apheresis, patients underwent tetanus vaccination (if no tetanus booster had been given in the past year). Four weeks after undergoing apheresis, patients initiated FRα-loaded DC vaccine treatment. Patients were treated with five cycles of induction vaccination, given every 3 weeks (1–2 × 10^7^ DCs/dose). Patients underwent disease evaluation via history, exam, and CA-125 prior to cycle 4 (13 weeks after apheresis). Patients with CA-125 elevations as the only sign of recurrence could remain on the protocol until an additional CA-125 level confirmed biochemical recurrence. Patients who completed the induction phase of DC vaccination without confirmation of recurrence underwent repeat disease evaluation and maintenance DC vaccination every three months for up to two years from study enrollment. Patients could receive up to 12 doses of vaccine therapy.

At the trial onset, the vaccine was administered intradermally to two ipsilateral areas of the body (proximal arm/deltoid and anterior/lateral thigh in four 0.125 ml doses per area at least 3 cm apart through an intradermal needle (Becton Dickinson, Franklin Lakes, NJ). During the course of the trial, this intradermal needle was discontinued. Thereafter, DCs were administered intradermally to a single area (either proximal arm or thigh) via a 3 M hollow Microstructured Transdermal System (hMTS) device, a single device that delivers vaccine intradermally through multiple needles (3 M, Maplewood, MN). Each patient was treated via a single injection method throughout her treatment course.

Patients were monitored for toxicity using the Common Terminology Criteria for Adverse Events version 4.0 (CTCAE v 4.0) at baseline and three weeks after each cycle of treatment. Patients also underwent basic hematology and chemistry laboratory evaluations at baseline and three weeks after each cycle of treatment to assess for vaccine toxicity.

### Assessments of immunogenicity and immune cell profiling

Patients underwent large (200 ml) blood draws for research purposes on week 0 (pre-treatment), week 19 (post-induction), and either week 107 (post-maintenance) or at study discontinuation for recurrence, as well as smaller (25 ml) research blood draws prior to each cycle of therapy. Research blood samples were collected in sodium heparin tubes for PBMCs and plasma. Heparin tubes were processed for PBMCs and plasma using Ficoll density gradient centrifugation on the day of the blood draw, and cells were then slowly frozen to −80C and subsequently to <−196C in liquid nitrogen for batch analysis.

ELIspot assays were performed to quantitate antigen-specific T cells capable of secreting IL-17 and IFN-*γ*^25,59^. PBMCs were thawed and plated at 2 × 10^5^ PBMCs per well in triplicate in 96-well plates. Cells were incubated at 37 °C with medium alone, Cyclin D1 peptide (MELLLVNKLKWNLAA, negative control), FR30, FR56, FR76, FR113, FR238, FRα protein, tetanus toxoid (TT, positive control), or phytohemagglutinin (PHA, positive control)^59^. After 24 h, cells were transferred to nitrocellulose plates, coated with either anti-IFN-*γ* or IL-17 antibodies, and incubated for 24 more hours. Plates were then washed and incubated with biotinylated anti-IFN-*γ* or IL-17 antibodies, streptavidin-alkaline phosphatase, and colorimetric substrate, with washes between each step. After drying overnight, the plates were read on an AID ELIspot reader (San Diego, CA). Antigen-specific T cells were defined as the average number of spots elicited by the antigen of interest minus the average number of spots elicited when cells were incubated with culture medium alone, without the addition of any peptides.

Antibody responses were assessed with ELISA^25^. Ninety-six-well plates were coated with 20 ng/ml of FR30, FR56, FR113, FR238, or Cyclin D1 peptide, or with 100 ng/ml TT or FRα protein. Human IgG was used as a standard. Plates were blocked with 1% bovine serum albumin; then, patient plasma samples were added at a 1:100 dilution and incubated for 2 h at room temperature. Anti-human IgG-HRP (Santa Cruz Biotechnology, Santa Cruz, CA) was used at a dilution of 1:5000 to detect antigen-specific IgG. ELISA Substrate (BD Biosciences, Franklin Lakes, NJ) was added in standard fashion, and light absorbance at 450 nm was quantitated on a plate reader. Relative isotypes (IgG1, IgG2, IgG3, and IgG4) were also determined by ELISA. In brief, flat-bottom 96-well microplates were coated overnight at 4 °C with 100 μL/well of 0.05 M carbonate-bicarbonate buffer containing 100 ng/ml of FRα protein (Sino Biological). After washing with PBS containing 0.05% Tween 20, the wells were blocked with PBS containing 1% BSA. After washing, human plasma was added to the plate and incubated overnight at 4 °C. After washing, 100 μL/well of anti-human IgG subclass antibodies (anti-IgG1, anti-IgG2, anti-IgG3, and anti-IgG4) conjugated with HRP (Invitrogen) were diluted 1:1000 and incubated for 1 h at RT. After a final wash, each well was incubated with 100 μL TMB substrate (BD Bioscience). Color development was stopped by the addition of 50 μL/well of diluted HCL and absorbance was read at 450 nm on a plate reader. Isotype-specific antibody concentrations were determined using a subclass-specific IgG standard curve done in parallel.

Tregs were quantitated by mass cytometry. In brief, PBMCs were thawed, incubated at 37 °F for 48 h with either culture medium alone or FRα protein, then labeled with metal-conjugated antibodies to CD45 (1:400), CD196 (CCR6, 1:200), IL-4 (1:100), CD5 (1:200), CD4 (1:200), IL-6 (1:200), CD278 (inducible T-cell costimulatory—ICOS), CD25 (IL-2 receptor alpha—IL-2Rα, 1:200), IL-5 (1:100), tumor necrosis factor alpha (TNFα, (1:400), CD45RA (1:200), T-cell immunoglobulin and mucin-domain containing-3 (TIM-3, 1:200), PD-1 (1:100), CD183 (CXCR3, 1:200), CD194 (CCR4, 1:200), CD197 (CCR7, 1:200), CD28 (1:400), CD274 (PD-L1, 1:400), transforming growth factor beta (TGFβ, 1:100), IL-17a (1:400), CD45RO (1:100), IL-10 (1:200), CD27 (1:200), IFN-*γ* (1:200), CD19 (1:400), CD3 (1:200), CD185 (CXCR5, 1:100), granzyme B (1:100), CD8a (1:400), perforin (1:100), and CD127 (1:200). All antibodies were from Fluidigm. PBMCs were incubated with cell surface targeting antibodies for 45 min. After this, PBMCs were fixed with 2% paraformaldehyde, washed, and incubated with antibodies to intracellular antigens for 45 min. After labeling, PBMCs were assayed on a Helios II mass cytometer (Fluidigm, South San Francisco, CA). Data files were normalized with CyTOF software version 6.7.1014 (Fluidigm) and were subsequently analyzed using Gemstone Software (Verity Software House, Topsham, ME) to remove doublets, dead cells, and non-specific events. Immunophenotyping analysis to quantify Tregs was performed via the R package Cytofkit which includes cluster analysis using Rphenograph, ClusterX and FlowSOM (The R Project for Statistical Computing, Vienna, Austria).

### Immunohistochemistry and immunohistofluorescence

FRα expression levels in patient tumor samples were measured using previously established immunohistochemistry (IHC) methods^60,61^. In brief, formalin-fixed paraffin-embedded (FFPE) sections were heated (1 h, 60 °C) and deparaffinized in sequential baths of xylene, ethanol, and water. The slides were treated under pressure (125 °C at 16 psi for 30 s) with Diva heat-induced epitope retrieval solution (Biocare Medical) followed by blocking with Peroxidase-1 blocking solution (Biocare Medical) and a serum-free universal blocking reagent. After blocking, the slides were incubated with a 1:100 dilution of either anti-FRα murine monoclonal antibody (Clone 26B3.F2, Biocare Medical) or Bond Negative Mouse ready-to-use negative control antibody (Dako). The slides were then washed and incubated for 15 min. with MACH4 Mouse Probe Primary Antibody Enhancer (Biocare Medical) followed by 20 min. incubation with Universal Polymer-HRP reagent (Biocare Medical). For colorimetric antigen detection, the slides were stained with a 3,3’-diaminobenzidine tetrahydrochloride solution (Dako) followed by counter-staining with hematoxylin. Finally, the slides were washed, dehydrated with several changes of ethanol and xylene and coverslips were mounted. The slides were imaged and analyzed using the Aperio ScanScope Image Scanner and Aperio ImageScope software v12.4.2.7000 (Aperio Technologies). FRα expression membrane staining was quantified as follows: negative (0), weak (1+), moderate (2+) and strong (3+) membrane staining. The percentages of cells stained at each intensity were recorded to calculate an H score which is a weighted score that captures both the proportion of positive staining and its intensity^60^. The final score for staining each sample was defined as: H-score = 0* (% at 0) + 1* (% at 1+) + 2* (% at 2+) + 3* (% at 3+), where 300 is the highest possible score.

IHC for CD8 and PDL1 was done on FFPE slides that were deparaffinized and rehydrated and treated with Target Retrieval pH 6.0 (Dako #S1699) for 25 min at 99 °C. The slides were cooled in the solution for 25 min and washed three times with TBST (Dako #S3006). Endogenous peroxidase was quenched by treating the slides with 3% H_2_O_2_ for 5 min at room temperature. The slides were washed and treated with Serum-Free Protein Block (Dako) for 5 min at room temperature. The primary antibodies (PD-L1, Invitrogen and CD8, Novus Biologicals) were applied to the slides at a dilution of 1:100 for 1 h at room temperature. After washing away the excess unbound primary antibody, the secondary antibody (anti-rabbit labeled polymer—Dako) was applied to the slides for 30 min at room temperature. The slides were washed with TBST and treated with DAB+(Dako K3468) for 5 min at room temperature. The stained slides were washed with distilled water and counterstained with Gills I hematoxylin, dehydrated and mounted. Analysis of CD8 T cell infiltration was done using digital quantification of 20 fields with the Aperio ScanScope image scanner. The fields were selected based on the abundant expression of the intraepithelial tumor-infiltrating lymphocytes. The final score, representing CD8 T cells, was derived from the mean of the scores obtained the selected fields.

PD-L1 expression was quantified based on the percentage of the tumor cells expressing a positive signal. PD-L1 negative expression was designated a score of zero. PD-L1 weak positive (score of 1) is defined as membranous PD-L1 expression in 1–25% of tumor cells, and PD-L1 moderate expression is defined as membrane expression in 26% to 50% of tumor cells (score of 2). PD-L1 strong expression (score of 3) is defined as membrane expression >50% of tumor cells.

Immunofluorescence for intratumoral Treg infiltration was performed using FFPE tissue slides. The tissue slides were deparaffinized and hydrated through graded alcohols and water. The slides were treated with Target Retrieval pH 9.0 (Dako #2367) for 25 min at 99 °C. Following antigen retrieval, the slides were cooled in the solution for 25 min, rinsed with TBST (Dako #S3006). The endogenous peroxidase activity was quenched with 3% H_2_O_2_ for 5 min at room temperature. The slides were rinsed with TBST and blocked with Serum-Free Protein Block (Dako #X0909) for 5 min at room temperature. The slides were incubated with the primary antibodies CD4 (Abcam) at 1:25; CD25 (Novocastra) at 1:25; and FoxP3 (R&D systems) at 1:30 for 1 h at room temperature followed by two changes of TBST. After removing the excess primary antibodies, the slides were incubated with the secondary antibodies Alexa-Fluor anti-goat 568, Alexa-Fluor anti-mouse 488, and Alexa-Fluor anti-rabbit 647 (all at 1:250 dilution) for 30 minutes at room temperature followed by two changes in TBST. The specimens were treated with DAPI at room temperature for 10 min. The slides were mounted with aqueous mounting media, covered with coverslip and sealed. The slides thus stained were imaged at ×20 magnification using Zeiss LSM 880 confocal microscope. The laser settings were optimized and all the slides were imaged with the same setting. Twenty fields were imaged for each sample (except for four specimens that were too small); the cells that were positive for CD4, CD25, and FoxP3 were quantified for each field; and the average or total number of triple-positive cells per specimen was calculated.

### Antibody-dependent cellular cytotoxicity (ADCC) assay

IGROV1 ovarian tumor cells (gift from Dr. Michael Barry, Mayo Clinic) were labeled with PKH26 (Sigma Aldrich, St. Louis, MO). Patient post-immunization plasma was heat inactivated for 30 min at 56 °C and then mixed with 2 × 10^5^ tumor cells in RPMI with 10% FBS followed by incubation at 37 °C for 45 min. After incubation, magnetically purified NK cells (Miltenyi Biotech, Somerville, MA), derived from discarded de-identified apheresis cones were added to the tubes at a 1:5 IGROV1:NK ratio and incubated at 37 °C for 4 h. The cells were washed and stained with fixable AF450 viability dye (eBioscience, Carlsbad, CA) followed by washing and fixing with 1% paraformaldehyde. Live and dead tumor cells were analyzed using standard flow cytometry.

### Statistical methods

Data for analysis were collected during the course of recruitment, throughout the treatment period and throughout the follow-up period. All clinical data were collected in a standard outpatient setting at the Mayo Clinic in Rochester, MN. Laboratory data, including levels of antigen-specific T cells, intratumoral CD8 T cells, PD-L1-positive tumor cells, FRα-positive tumor cells, Tregs, and antibodies were collected either in Dr. Knutson’s laboratory at the Mayo Clinic in Jacksonville, FL, Dr. Block’s laboratory in Rochester, MN, or in Dr. Pavelko’s laboratory in Rochester, MN. The primary clinical endpoint was the percentage of patients who developed a severe toxicity (Grade 3–5 AE, NCI-CTCAE version 4.0). Initially, it was determined that the vaccine treatment would be considered safe if fewer than 5 of a planned 22 accrued patients developed DLT. The sample size of 22 patients was chosen based on our previous studies using other vaccine approaches^25^. The study was terminated at 19 patients as it was deemed safe based on complete absence of grade 3 or higher toxicity in all of the treated patients. RFS and OS were defined as time from registration to disease recurrence (either radiologic of confirmed CA-125 levels) and estimated with the Kaplan and Meier method^62^. The laboratory endpoints for the reported analysis herein were levels of antigen-specific T cells, CD8 T cells, PD-L1+ cells, FRα-positive tumor cells Tregs, antibodies which were compared using Graphpad software (Instat, V.3. and Prism, V.8., GraphPad, San Diego CA). A patient was considered to have immunologically responded if she had developed a ≥3-fold increase in FRα-specific T cells or a ≥2-fold increase antibodies at any point during the vaccine period^25,63^. If baseline T cell immunity was undetectable, a positive response was defined as ≥50 antigen-specific T cells/million PBMC. Pre- and post-immunization T cell and antibody responses were compared using the either the nonparametric Wilcoxon matched pairs test or the paired Student’s *T* test corrected, where appropriate, for multiple comparisons using the Bonferroni or Benjamini-Hochberg methods. Correlations were examined using either the Pearson’s or Spearman’s correlations test depending on the normal distribution of the residuals. Time course data were compared using two-way analysis of variance. Best-fit lines, for data visualization purposes only were calculated using the nonlinear least-squares method. Testing was done with two-sided or one-sided tests and *p* ≤ 0.5 was considered significant as indicated in text or legends.

### Reporting summary

Further information on research design is available in the Nature Research Reporting Summary linked to this article.

## Supplementary information

Supplementary Information

Reporting Summary

## Data Availability

Data supporting all the figures and tables in this article are not publicly available in order to protect patient privacy, but any and all of the data can be made available on reasonable request from the corresponding author at any time in a deidentified manner. The corresponding author will evaluate whether the completion of a Data Usage Agreement is required. Corresponding author details are: Dr. Keith L. Knutson, Department of Immunology, Mayo Clinic Jacksonville, email: knutson.keith@mayo.edu.
