# Supplementary material for: Th17-inducing autologous dendritic cell vaccination promotes antigen-specific cellular and humoral immunity in ovarian cancer patients
Source: Nat Commun. 2020 Oct 14;11:5173. doi: 10.1038/s41467-020-18962-z (PMC7560895; doi:10.1038/s41467-020-18962-z)

Supplementary Information

Title: Th17-inducing autologous dendritic cell vaccination promotes antigen-specific cellular and humoral immunity in ovarian cancer patients

Authors: Matthew S. Block[1], Allan B. Dietz[2], Michael P. Gustafson[2], Kimberly R. Kalli[1], Courtney L. Erskine[3], Bahaaeldin Youssef[4], Geraldine V. Vijay[4], Jacob B. Allred[5], Kevin D. Pavelko[3], Michael A. Strausbauch[6], Yi Lin[7], Megan E. Grudem[1], Aminah Jatoi[1], Carolyn M. Klampe[1], Andrea E. Wahner-Hendrickson[1], S. John Weroha[1], Gretchen E. Glaser[8], Amanika Kumar[8], Carrie L. Langstraat[8], Mary L. Solseth[2], Michael C. Deeds[2], Keith L. Knutson[4]* and Martin J. Cannon[9]*.

Affiliations: [1]Department of Oncology, Mayo Clinic, Rochester, MN 55905, [2]Department of Laboratory Medicine and Pathology, Mayo Clinic, Rochester, MN 55905, [3]Department of Immunology, Mayo Clinic, Rochester, MN 55905, [4]Department of Immunology, Mayo Clinic, Jacksonville, FL 32224, [5]Mayo Clinic Cancer Statistics, Mayo Clinic, Rochester, MN 55905, [6]Immune Monitoring Core, Mayo Clinic, Rochester, MN 55905, [7]Department of Medicine, Mayo Clinic, Rochester, MN 55905, [8]Department of Obstetrics and Gynecology, Rochester, MN 55905, [9]Department of Microbiology and Immunology, University of Arkansas for Medical Sciences, Little Rock, AR 72205

*These authors jointly supervised this work

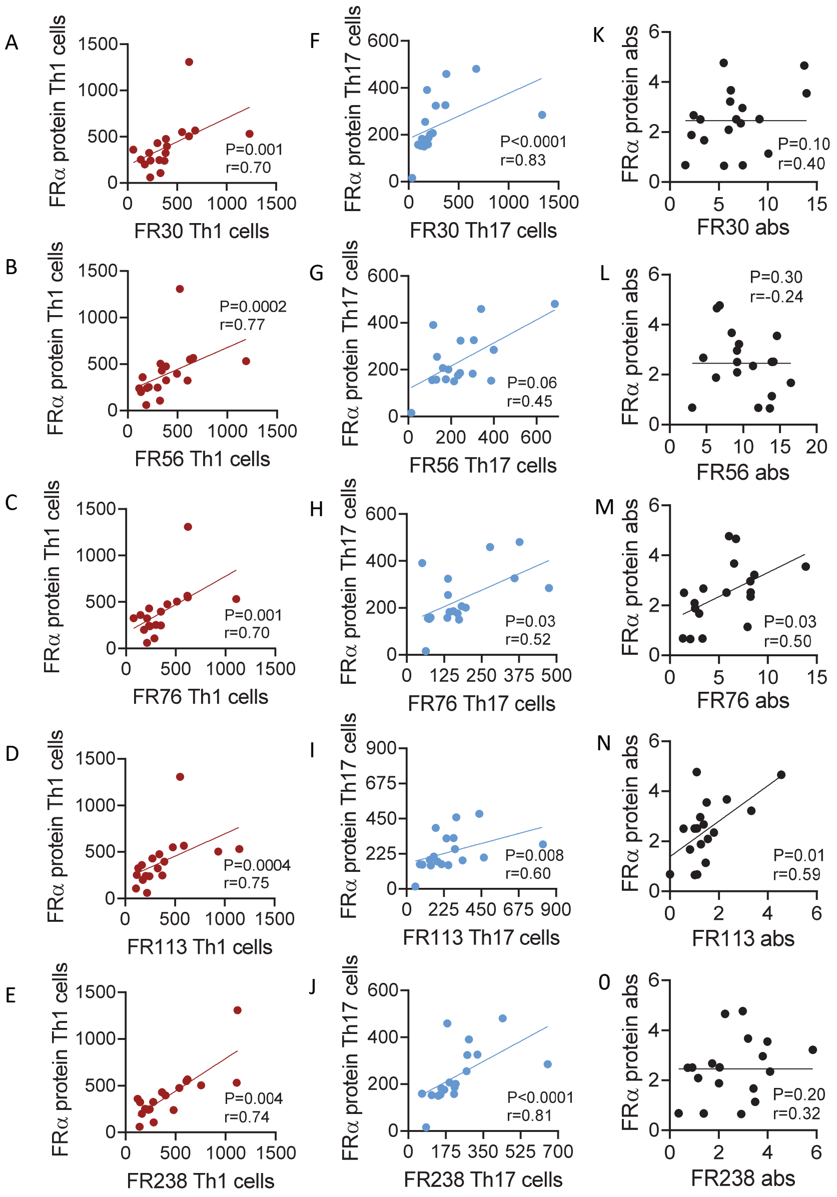

Supplementary Fig. 1: Generation of IFN-γ^+ T cell, IL-17$^+$ T cell and humoral immunity to whole FRα protein is linked to generation of immunity to vaccine peptides. Shown in **Panels A-E** are correlation analyses comparing the magnitude of maximal peptide-specific IFN-γ^+ T cell response to the maximal FRα IFN-γ^+ response. Each panel represents a unique peptide and each symbol represents a unique patient (n=18). **Panels F-J** and **Panels K-O** show the same for IL-17$^+$ T cell and antibody responses, respectively. r = spearman correlation rho coefficient and unadjusted 2-sided P values indicate significant deviation from zero slope. Inset best-fit lines were calculated using non-linear least squares regression for data trend visualization.

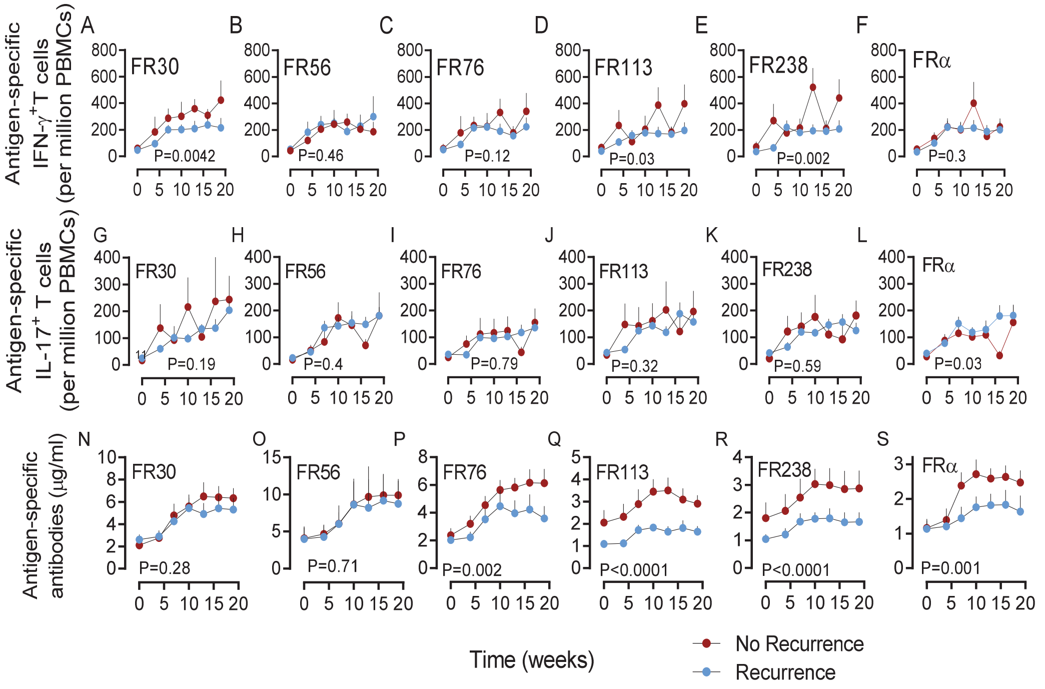

Supplementary Fig. 2: Vaccine-induced immunity correlates with disease recurrence. Panels A-F, **Panels G-L**, and **Panels N-S** compare the mean (+ s.e.m.) Th1, Th17, and antibody immune responses, respectively, of those who recurred (blue symbols, n=11) and those who did not recur (red symbols, n=7). P values, comparing separation of the 2 curves are calculated using a 2-sided, 2-way ANOVA test.

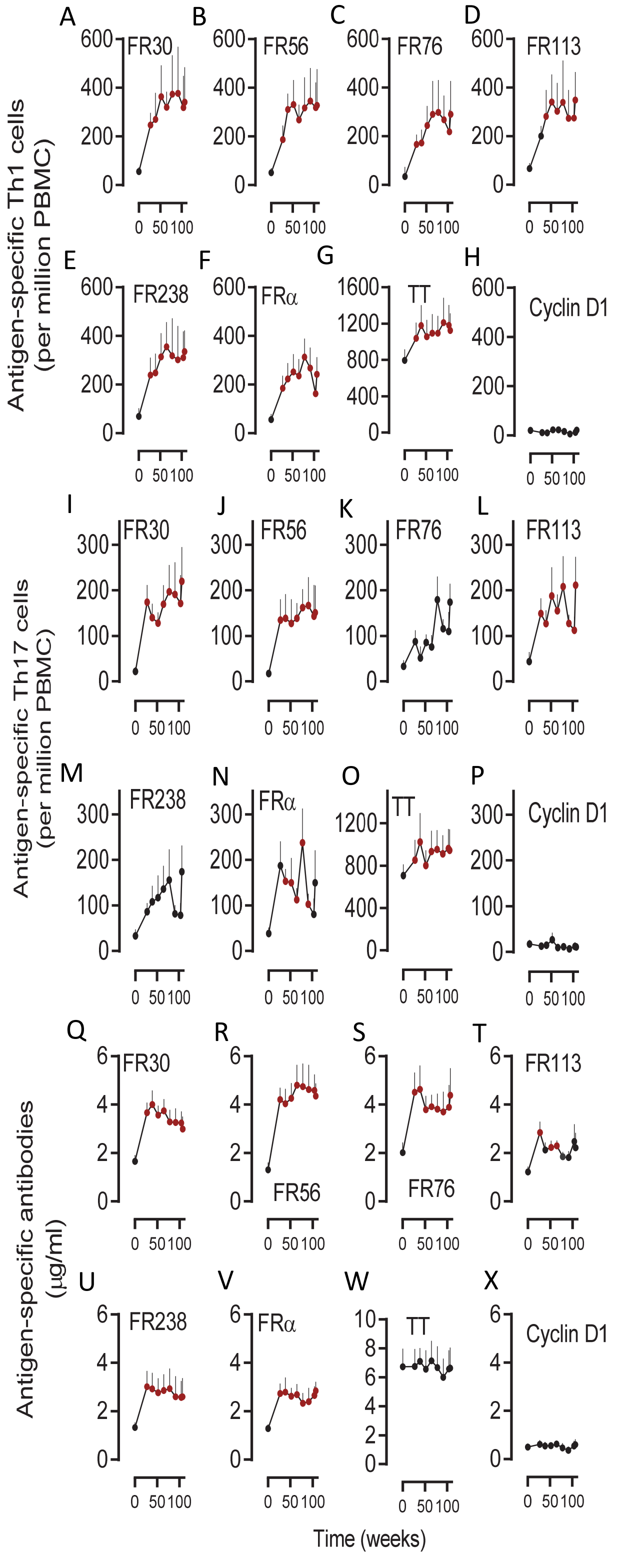

Supplementary Fig. 3: T cell and antibody immunity persisted for at least 2 years. Panels A-H and **I-P** are maintenance phase time courses of antigen-specific IFN-γ^+ T cells (A-H) and IL-17^+ (I-P) T cell frequencies (T cells per million PBMCs, mean + s.e.m.) for FRα peptides FR30, FR56, FR76, FR113 and FR238 FRα protein, TT, and control cyclin D1 peptide, respectively, in 8 evaluable patients. Shown in **Panels Q-X** are maintenance phase time courses of antigen-specific antibody frequencies (μg/ml, mean + s.e.m.). Reddened symbols indicate Benjamini-Hochberg-corrected P≤0.05 significance calculated with the one-sided Wilcoxon matched pairs test, compared with baseline responses to that antigen. Exact P values are indicated in Supplementary Tables 9-11.

Supplementary Table 1: Summary of FRα DC vaccine manufacturing results

Patient #	# of leukapheresis collections	Doses manufactured	Cells/dose (in millions)	Release criteria met (Y/N)
001	1	23	20	Y
002	1	16	20	Y
003	1	28	20	Y
004	1	23	20	Y
005	1	20	20	Y
006	1	24	20	Y
007	1	30	20	Y
008	1	24	20	Y
009	1	12	12	Y
010	2	20	20	N
		5	10	Y
011	1	22	20	Y
012	1	12	12.5	Y
013	1	21	16	Y
014	1	12	15	Y
015	1	20	16	Y
016	1	33	16	Y
017	1	24	16	Y
018	1	21	16	Y
019	1	15	16	Y

Supplementary Table 2: Adverse events

Adverse Event	Grade			
	1		2	
**Type**	**N**	**%**	**N**	**%**
**Injection site reaction**	18	94.7		
**Arthralgia**	10	52.6	3	15.8
**Myalgia**	5	26.3		
**Pruritus**	4	21.1	1	5.3
**Bone pain**	4	21.1		
**Diarrhea**	3	15.8		
**Maculopapular rash**	2	10.5	1	5.3
**Fatigue**	1	5.3	1	5.3
**Neutrophil count decreased**	1	5.3	1	5.3
**Pain**	2	10.5		
**Platelet count decreased**	2	10.5		

All Grade 2+ Toxicities and all Grade 1+ Toxicities with at least 10% incidence are reported.

Supplementary Table 3: Wilcoxon matched pairs 2-sided P values versus time point 0 for Figure 1A-H

	**Time point**					
**Antigen**	**4 week**	**7 week**	**10 week**	**13 week**	**16 week**	**19 week**
**FR30**	0.0599	<0.0001	<0.0001	0.0001	<0.0001	<0.0001
**FR56**	0.0026	<0.0001	<0.0001	0.0001	0.0005	0.0017
**FR76**	<0.0001	<0.0001	<0.0001	0.0002	<0.0001	0.0005
**FR113**	0.0066	0.0079	0.0005	0.0001	0.0003	0.0001
**FR238**	0.0063	0.0002	0.0002	<0.0001	0.0003	<0.0001
**FRα protein**	0.0003	0.0014	0.0003	<0.0001	0.0011	<0.0001
**TT**	0.4951	0.0104	0.0182	0.0007	0.0047	0.0151
**Cyclin D1**	0.2958	0.3484	0.1876	0.2114	0.654	0.4548

Supplementary Table 4: Wilcoxon matched pairs 2-sided P values versus time point 0 for Figure 1I-P

	Time point					
**Antigen**	**4 week**	**7 week**	**10 week**	**13 week**	**16 week**	**19 week**
**FR30**	0.0181	0.0017	<0.0001	<0.0001	0.0008	<0.0001
**FR56**	0.0042	0.0023	<0.0001	<0.0001	<0.0001	<0.0001
**FR76**	0.2979	0.0139	0.0011	0.0034	0.0539	0.0003
**FR113**	0.1754	0.0017	0.0005	0.0013	0.0003	<0.0001
**FR238**	0.0129	0.0008	0.0002	0.0017	0.0027	0.0012
**FRα protein**	0.0008	0.0016	0.0002	0.0008	0.0040	0.0009
**TT**	0.5798	0.0077	0.0056	0.0023	0.0008	0.0006
**Cyclin D1**	0.8900	0.8999	0.9999	0.9341	0.4973	0.1531

Supplementary Table 5: Benjamini-Hochberg adjusted 2-sided P values for Figure 2G

	**FR30**	**FR56**	**FR76**	**FR113**	**FR238**	**FRα protein**
**FR30**		0.0004	<0.0001	<0.0001	<0.0001	0.0013
**FR56**	0.0004		0.0076	0.0055	0.0076	0.0010
**FR76**	<0.0001	0.0076		0.0002	<0.0001	0.0016
**FR113**	<0.0001	0.0055	0.0002		0.0006	0.0004
**FR238**	<0.0001	0.0076	<0.0001	0.0006		0.0005
**FRα protein**	0.0013	0.0010	0.0016	0.0004	0.0005	

Supplementary Table 6: Benjamini-Hochberg adjusted 2-sided P values for Figure 2H

	FR30	FR56	FR76	FR113	FR238	FRα protein
**FR30**		0.0050	0.0040	0.0044	0.0028	0.0001
**FR56**	0.0050		0.0040	0.0049	0.1496	0.0771
**FR76**	0.0004	0.0040		0.0088	0.0268	0.0322
**FR113**	0.0044	0.0049	0.0088		0.0399	0.0103
**FR238**	0.0028	0.1496	0.0268	0.0399		0.0002
**FRα protein**	<0.0001	0.0771	0.0322	0.0103	0.0002	

Supplementary Table 7: Wilcoxon matched pairs 2-sided P values versus time point 0 for Figure 3A-H

	**Time point**					
**Antigen**	**4 week**	**7 week**	**10 week**	**13 week**	**16 week**	**19 week**
**FR30**	0.0047	0.0003	<0.0001	0.0001	<0.0001	<0.0001
**FR56**	0.0066	<0.0001	<0.0001	<0.0001	<0.0001	<0.0001
**FR76**	0.0034	<0.0001	<0.0001	<0.0001	<0.0001	<0.0001
**FR113**	0.1540	<0.0001	<0.0001	<0.0001	<0.0001	0.0021
**FR238**	0.0155	<0.0001	<0.0001	<0.0001	0.0005	<0.0001
**FRα protein**	0.0887	0.0005	<0.0001	0.0002	<0.0001	<0.0001
**TT**	0.0599	0.1591	0.0174	0.0714	0.1743	0.0021
**Cyclin D1**	0.7987	0.8900	0.0034	0.0267	0.2069	0.2439

Supplementary Table 8: Benjamini-Hochberg-adjusted 2-sided P values for Figure 3N

	FR30	FR56	FR76	FR113	FR238	FRα protein
**FR30**		0.1613	0.0008	0.0418	0.1563	0.1245
**FR56**	0.1613		0.3882	0.5789	0.3606	0.4139
**FR76**	0.0008	0.3882		0.1137	0.0064	0.0462
**FR113**	0.0418	0.5789	0.1137		0.0944	0.0503
**FR238**	0.1563	0.3606	0.0064	0.0944		0.2517
**FRα protein**	0.1245	0.4139	0.0462	0.0503	0.2517	

Supplementary Table 9: : Benjamini-Hochberg adjusted Wilcoxon matched pairs 1-sided P values versus time point 0 for Supplementary Figure 3A-H

	Time point							
**Antigen**	**27 week**	**39 week**	**52 week**	**65 week**	**78 week**	**91 week**	**104 week**	**107 week**
**FR30**	0.0312	0.0156	0.0156	0.0156	0.0104	0.0178	0.0125	0.0193
**FR56**	0.0223	0.0312	0.0312	0.0250	0.0312	0.0208	0.0416	0.0234
**FR76**	0.0312	0.0313	0.0156	0.0178	0.0156	0.0156	0.0125	0.0208
**FR113**	0.0624	0.0208	0.0208	0.0391	0.0312	0.0245	0.0156	0.0312
**FR238**	0.0312	0.0313	0.0156	0.0156	0.0125	0.0178	0.0104	0.0208
**FRα protein**	0.0312	0.0358	0.0208	0.0391	0.0156	0.0208	0.0156	0.0250
**TT**	0.0156	0.0178	0.0156	0.0391	0.0312	0.0124	0.0312	0.0208
**Cyclin D1**	0.4821	0.8126	0.4727	0.5625	1.0000	0.9168	1.0000	0.6501

Supplementary Table 10: : Benjamini-Hochberg adjusted Wilcoxon matched pairs 1-sided P values versus time point 0 for Supplementary Figure 3I-P

	Time point							
**Antigen**	**27 week**	**39 week**	**52 week**	**65 week**	**78 week**	**91 week**	**104 week**	**107 week**
**FR30**	0.0312	0.0156	0.0156	0.0125	0.0104	0.0178	0.0104	0.0156
**FR56**	0.0178	0.0156	0.0312	0.0250	0.0156	0.0208	0.0208	0.0156
**FR76**	0.0729	0.5000	0.0728	0.2143	0.0780	0.0626	0.0782	0.1248
**FR113**	0.0312	0.0358	0.0520	0.0374	0.0390	0.0547	0.0312	0.0312
**FR238**	0.1564	0.1459	0.1563	0.1484	0.1429	0.2083	0.1562	0.1248
**FRα protein**	0.0624	0.0501	0.0390	0.0521	0.0468	0.0416	0.1875	0.0625
**TT**	0.0156	0.0178	0.0156	0.0391	0.0312	0.0125	0.0312	0.0208
**Cyclin D1**	0.5000	0.4167	0.3929	0.8752	0.4688	0.6252	0.5835	0.5626

Supplementary Table 11: : Benjamini-Hochberg adjusted Wilcoxon matched pairs 1-sided P values versus time point 0 for Supplementary Figure 3Q-X

	**Time point**							
**Antigen**	**27 week**	**39 week**	**52 week**	**65 week**	**78 week**	**91 week**	**104 week**	**107 week**
**FR30**	0.0312	0.0250	0.0156	0.0104	0.0156	0.0208	0.0178	0.0156
**FR56**	0.0208	0.0156	0.0312	0.0156	0.0125	0.0104	0.0089	0.0078
**FR76**	0.0312	0.0178	0.0156	0.0104	0.0125	0.0234	0.0208	0.0156
**FR113**	0.0312	0.0626	0.0312	0.0208	0.0729	0.1094	0.0626	0.0625
**FR238**	0.0312	0.0250	0.0156	0.0104	0.0156	0.0547	0.0208	0.0447
**FRα protein**	0.0312	0.0156	0.0156	0.0104	0.0125	0.0178	0.0104	0.0156
**TT**	0.500	0.4501	0.5468	0.6147	0.3855	0.3929	0.8752	0.3438
**Cyclin D1**	0.3333	0.4376	0.6501	0.5468	0.1136	0.4688	0.5417	0.4822

Supplementary Table 12: FRα peptides used for FRα DC vaccine

Single letter amino acid sequence	Positions	Designation	Length
RTELLNVCMNAKHHKEK	30-46	FR30	17
QCRPWRKNACCSTNT	56-70	FR56	15
KDVSYLYRFNWNHCGEMA	76-93	FR76	18
LGPWIQQVDQSWRKERV	113-129	FR113	17
PWAAWPFLLSLALMLLWL	238-255	FR238	18

Mayo Clinic Cancer Center

MC1361, A pilot study of the safety and immunogenicity of folate receptor alpha peptide-loaded dendritic cell vaccination in patients with advanced stage epithelial ovarian cancer

Study Chairs: Matthew S Block MD PhD
Mayo Clinic
200 First Street SW
Rochester, MN 55905
[redacted]

Keith L Knutson PhD√
Mayo Clinic
4500 San Pablo Blvd
Jacksonville FL 32224

Martin J Cannon, PhD√
Division of Gynecologic Oncology, Department of Obstetrics and Gynecology
University of Arkansas for Medical Sciences
4301 West Markham
Little Rock, AR 72205, USA
[redacted]

Allan B Dietz PhD √
Mayo Clinic
200 First Street, SW
Rochester, MN 55905
[redacted]

Statistician: Jacob (Jake) B Allred MS√

Drug Availability

Drug Company Supplied: *Agent(s): None*

√Study contributor(s) not responsible for patient care.

Document History	(Effective Date)
Activation	April 14, 2014
MCCC Addendum 1	April 14, 2014
MCCC Addendum 2	May 29, 2014
MCCC Addendum 3	April 23, 2015
MCCC Addendum 4	April 19, 2016
MCCC Addendum 5	December 22, 2016
MCCC Addendum 6	May 6, 2019

Protocol Resources

. Questions:	Contact Name:
Patient eligibility*, test schedule, treatment delays/interruptions/adjustments, dose modifications, adverse events, forms completion and submission	Sharon F Solinger, Quality Assurance Specialist II<br>Phone: [redacted]<br>E-mail [redacted]
Forms completion and submission	Janet L Lensing, Clinical CRA<br>[redacted]<br><br>Theresa L McCabe Woollard, Data CRA<br>[redacted]
Protocol document, consent form, regulatory issues	See Protocol Catalog for current RPS assignment:<br>http://ccswww:8686/catalog/catalog/styDetail.jsf?sty_node=8087
Serious Adverse Events)	Patricia G. McNamara, SAE C<br>[redacted]

*No waivers of eligibility per NCI

Table of Contents

MC1361, A pilot study of the safety and immunogenicity of folate receptor alpha peptide-loaded dendritic cell vaccination in patients with advanced stage epithelial ovarian cancer 1

Protocol Resources..... 2

Table of Contents..... 3

Schema..... 4

1.0 Background..... 5

2.0 Goals..... 15

3.0 Patient Eligibility..... 16

4.0 Test Schedule..... 19

5.0 Grouping Factors: None..... 20

6.0 Registration/Randomization Procedures..... 20

7.0 Protocol Treatment..... 21

8.0 Dosage Modification Based on Adverse Events..... 22

9.0 Ancillary Treatment/Supportive Care..... 24

10.0 Adverse Event (AE) Reporting and Monitoring..... 25

11.0 Treatment Evaluation..... 29

12.0 Descriptive Factors..... 29

13.0 Treatment/Follow–up Decision at Evaluation of Patient..... 30

14.0 Body Fluid Biospecimens..... 31

15.0 Drug Information..... 35

16.0 Statistical Considerations and Methodology..... 38

17.0 Pathology Considerations/Tissue Biospecimens..... 42

18.0 Records and Data Collection Procedures..... 44

19.0 Budget..... 45

20.0 References..... 45

Appendix I ECOG PERFORMANCE STATUS..... 48

Appendix II INJECTION SITE RECORD..... 49

Appendix III Clinical Evaluation for Autoimmunity..... 50

Appendix IV Patient Measurement of DTH Reactions..... 51

Appendix V Instruction Page for Patient Measurement of (delayed-type hypersensitivity) DTH Skin Reactions..... 52

Schema

Prior to discussing protocol entry with the patient, call the MCCC Registration Office [redacted] to insure that a place on the protocol is open to the patient.

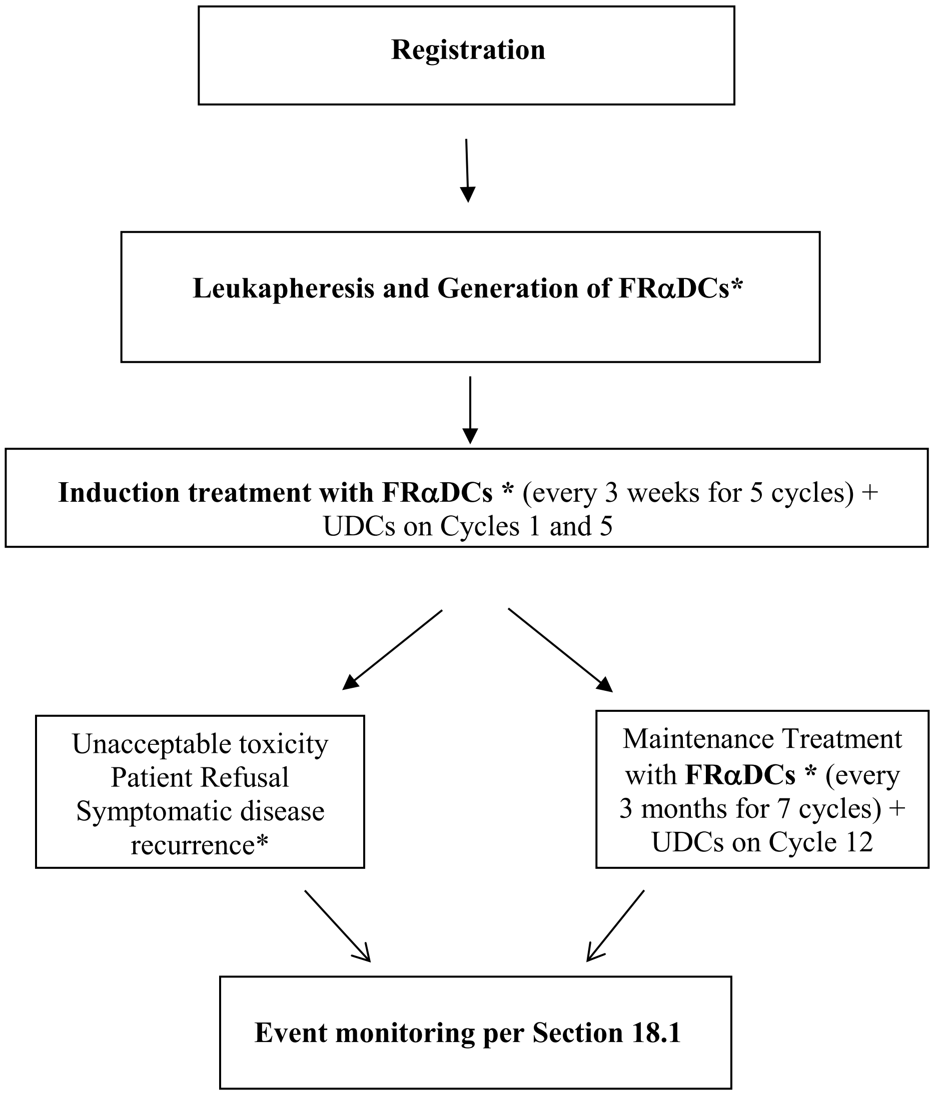

*** If at any time, patient experiences unacceptable toxicity, or refuses continued participation, then she will be placed on Event monitoring. Patients with asymptomatic disease recurrence may continue protocol treatment at the discretion of the treating investigator.**

Generic name: Folate Receptor Alpha-loaded dendritic cells<br>Brand name(s): None<br>Mayo Abbreviation: FRαDCs<br>Availability: Mayo Department of Laboratory Medicine and Pathology	Generic name: Unloaded dendritic cells<br>Brand name(s): None<br>Mayo Abbreviation: UDCs<br>Availability: Mayo Department of Laboratory Medicine and Pathology

1.0 Background

1.1 Need for novel adjuvant therapies for ovarian cancer

Ovarian cancer is the most lethal gynecologic malignancy, with a 5-year survival rate of 45% due to the fact that it is rarely diagnosed while the cancer is still localized [1]. After initial surgical debulking, standard chemotherapeutic agents used include platinum/ taxane combinations. Nearly 80% of patients with advanced stage disease respond to these drugs [1]. However, at least 70% of patients with an initial complete clinical response to treatment will subsequently experience recurrent disease. While second-line treatments are available and widely used, once ovarian cancer recurs it is not generally considered curable. Maintenance chemotherapies after completion of initial postoperative chemotherapy are occasionally prescribed, but no regimen has been shown to extend overall survival or improve the cure rate. Novel adjuvant strategies to prevent or delay recurrence of ovarian cancer are desperately needed.

1.2 Role of the immune system in the clinical course of ovarian cancer

Many studies have demonstrated the importance of the immune system in ovarian cancer patient outcome. Notably, Zhang and colleagues demonstrated that CD3 T cell infiltration was positively associated with survival [2]. These investigators also found that patients with tumor-infiltrating T cells were more likely to be optimally debulked at surgery, suggesting that T cell infiltration may directly limit disease spread.

Despite strong evidence for anti-tumor immunity, it has become increasingly apparent that ovarian tumors avail themselves of multiple mechanisms of immune evasion, the most prominent of which is recruitment and infiltration of regulatory T cells that suppress anti-tumor immunity. Regulatory T cells (Treg) are recruited to ovarian tumors by the chemokine CCL22 (predominantly expressed by ovarian tumors), and the presence of Treg confers immune privilege and is associated with a poor prognosis and increased mortality [3]. Other investigators have corroborated these observations, showing that high expression of the forkhead box transcription factor Foxp3, which is preferentially expressed by $CD4^+$ Treg, is an independent prognostic factor for reduced overall survival in ovarian cancer [4], and that a high $CD8^+$ T cell/Treg ratio is associated with a more favorable prognosis for this disease [5]. Further mechanisms that contribute to the immunosuppressed state include expression of B7-H1, which can promote T cell anergy and apoptosis through engagement of PD-1 expressed by effector T cells [6, 7] and expression of indoleamine 2,3-dioxygenase (IDO). Expression of both B7-H1 and IDO are associated with differentiation and recruitment of Treg [8-10], and clinical studies have shown that each of these mechanisms correlates independently with increased morbidity and mortality in ovarian cancer patients [11-13].

1.3 Th17-based immunotherapy and vaccination

In contrast with the evidence that Treg infiltration is associated with poor outcomes in ovarian cancer, a counterpoint is furnished by the recent observation that Th17 T cell infiltration correlates with more favorable clinical outcomes [14] (manuscript included in Section 8). Tumor-infiltrating Th17 cells were positively associated with effector cells and negatively associated with Treg infiltration [14], with the latter relationship arguably being founded on the known reciprocal regulation of Treg and Th17 differentiation [15, 16]. These observations have led to the question of whether Th17 cells could be induced

or expanded to therapeutic advantage, either by tumor vaccines or adoptive immunotherapy [17] (manuscript included in Section 8).

Current knowledge of the immunopathology of ovarian cancer presents a strong case in favor of Th17-based anti-tumor immunotherapy. This point is further supported by a recent report showing that human Th17 T cells are long-lived effector memory cells with the capacity to mediate effective antitumor immunity in collaboration with CD8 T cells. It was also found that Th17 cells were relatively resistant to apoptosis, and that apoptosis and persistence were regulated by high expression of HIF-1α, suggesting that Th17 cells may have a survival advantage in the hypoxic tumor microenvironment.

1.4 Dendritic cell vaccination in ovarian cancer

Dendritic cells (DCs) are myeloid-lineage immune cells and are primarily responsible for presenting antigens to T cells during the initiation of an adaptive immune response. Although DC vaccination has been tested for the treatment of many other malignancies, clinical studies of DC vaccination in ovarian cancer have been limited. DC pulsed with killed autologous primary ovarian tumor cells induced antigen-specific T cells that secreted IFNγ upon stimulation with autologous tumor cells [18], suggesting that antigen-pulsed DC may be a viable option for therapeutic vaccination against ovarian cancer. Various reports showing that DC loaded with tumor lysates, DC pulsed with acid-eluted peptides from ovarian cancer cells, DC fused with ovarian tumor cells, or DC loaded with ovarian tumor cells killed by oxidation could induce HLA class I-restricted CTL responses against autologous ovarian tumor cells [19-22] support this position. A limitation of these approaches is that the identity of the tumor antigens recognized by DC-stimulated CTL is not well defined, and it is not clear that T cell responses retain specificity for the tumor.

A phase I trial of autologous tumor antigen-loaded DC vaccination in 6 patients with ovarian cancer revealed no significant toxicity and 3 of 6 patients showed stable disease lasting 25 to 45 weeks [23]. Lymphoproliferative responses to tumor antigen were detected in 2 patients. Follow-up CT at 5 months after the last vaccination showed a partial response, and CT at 16 months showed greater than 50% remission of lymph node metastases. CA-125 levels were greatly reduced after the 1st vaccination (from 640 U/mL to 60 U/mL) and remained at baseline 11 months after completion of vaccination. A clinical trial of MUC1 and HER2/neu peptide-pulsed DC vaccination in patients with advanced ovarian or breast cancer reported peptide-specific CTL responses in 5 of 10 patients, and also showed evidence of epitope spreading [24]. In one patient vaccinated with MUC1 peptides, carcinoembryonic antigen and MAGE3 peptide-specific T-cell responses were detected, and in a second patient, MUC1-specific T-cell responses were detected after seven vaccinations with HER2/neu peptide-pulsed DC.

The most durable clinical response to DC vaccination was described in a case report of a patient with recurrent metastatic ovarian cancer, who received 10 vaccinations of autologous DC loaded with mRNA encoding folate receptor-α [25].

1.5 The folate receptor alpha as a vaccine target antigen

The tumor antigen targeted by the DC vaccine in this protocol is folate receptor alpha (FRα), a high affinity folate-binding protein that is overexpressed on 70-90% of ovarian

tumors [26]. It is expressed at low levels in a small number of other tissues in the body where it functions to move folic acid from one compartment to another. For example, in the kidneys, FRα is responsible for retrieving folate from the urine prior to its excretion; in the central nervous system, FRα appears to concentrate folate in cerebrospinal fluid. It has recently been shown that FRα–expression is common in metastatic foci present at the time of diagnosis of ovarian cancer as well as on recurrent tumors, indicating that FRα is a good tumor target regardless of whether the patient is newly diagnosed or experiencing disease recurrence [27] (manuscript included in Section 8).

1.6 Pre-clinical human and mouse studies evaluating immunity to FR□

Identification of FRα-derived peptide epitopes

Since the FRα is a self-antigen, there is likely to be some level of tolerance to the antigen. Thus, in order to target the protein in vaccine strategies, it was necessary to define epitopes (i.e. antigenic fragments) to which there remained a T cell repertoire. We hypothesized that such epitopes could be identified in patients with either ovarian or breast cancer who would have recognized the antigen during the clinical course of their disease and generated an immune response. To do this, the sequence of human FRα was screened as described [28] for peptides predicted to bind HLA class II molecules using RANKPEP. Fourteen peptides (15-18 amino acids in length) predicted to bind to 3 or more HLA DR molecules were selected for further testing (**Figure 1**) (manuscript included in Section 8).

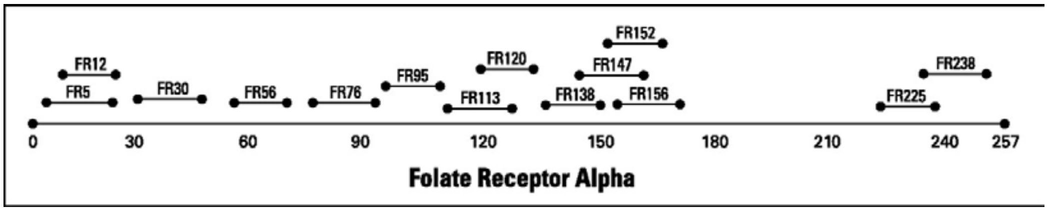

Figure 1: Figure shows the location of the fourteen potential epitopes along the FRα protein.

The peptides were chosen based on their ability to bind to several different HLA class II antigen presenting molecules. HLA class II antigens, expressed on antigen presenting cells, present peptides to CD4 T cells. The ability of the peptides to bind to multiple HLA class II molecules makes it likely that each patient would have antigen presenting molecules that could bind to some of the peptides, so the vaccine should be able to increase immune responses in most people. The peptides in Figure 1 were synthesized and peripheral bloods from 30 patients and 18 normal healthy volunteers were assessed for immune responses to each epitope and the whole protein using T cell ELIspot assays and antibody ELISAs. ELIspot analysis identified four peptides (FR30, FR56, FR 113, and FR238) that generated responses in more patients than in healthy donor counterparts (**Figure 2**). Overall, 70% of these patients showed FR-specific immunity to at least one epitope of FRα (**Figure 3**). This demonstrates that the ability to mount an immune response against FRα is intact and therefore should be able to be boosted.

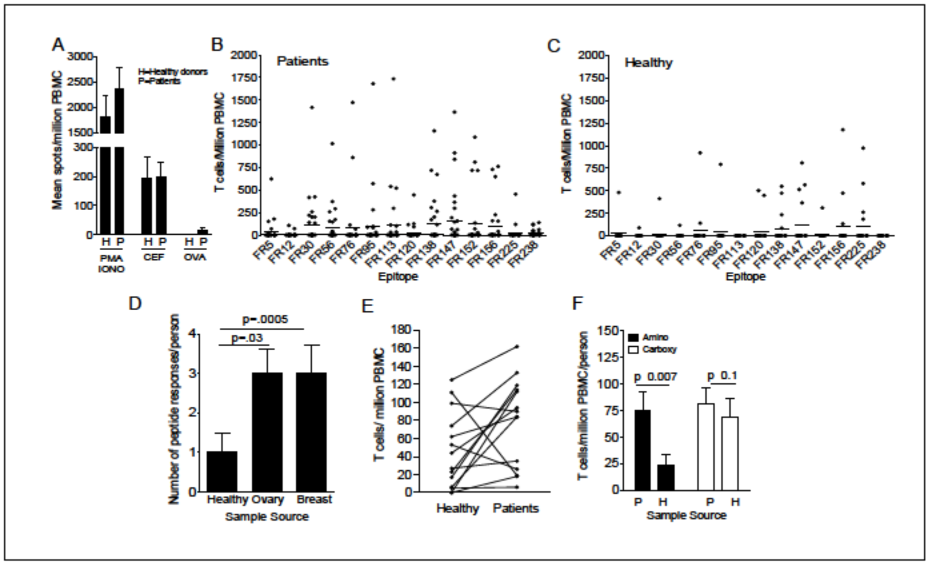

Figure 2. Patients generate immunity to multiple folate receptor alpha epitopes. Patients generate immunity to multiple folate receptor alpha (FR) epitopes. (A) Responses to controls. (B-C) T-cell frequencies (dot = one individual, bars = means). (D) Number of epitopes responded to by each group. (E) Relational diagram comparing frequencies of each peptide between groups. (F) Frequencies/person for amino and carboxy pools. H, healthy control; P, patient, PMA/IONO, phorbol myristate acetate/ionomycin; CEF, CMV, EBV and Flu peptides; OVA, ovalbumin peptide; PBMC, peripheral blood mononuclear cell; FITC, fluorescein isothiocyanate; APC, allophycocyanin; PEP, phycoerythrin.

AntFRα-specific antibodies and antibodies recognizing tetanus toxoid were assayed in sera using standard ELISAs against p76, a peptide that was predicted not only as a class II binding epitope but also an antibody epitope. Patients with previous breast or ovarian cancer had significantly higher levels of circulating anti-FRα antibodies than women who had never had cancer (**Figure 4**). Responses to tetanus toxin were similar in both groups. In conclusion, T cell responses and antibodies were evident for five peptides**, FR30, FR56, FR76, FR113, and FR238 (Table 1)**.

These peptides will form the basis of the DC vaccine and the sequences are shown in **Table I**. Detecting weak immune response in patients indicates that this part of the immune repertoire has not been deleted or anergized, so it should be able to be boosted through the use of DC vaccination. Interestingly, the sequence for FR238 contains an embedded HLA A2 (i.e. class I) epitope, allowing the potential for all components of a coordinated immune response – helper and cytotoxic T cells – to be engaged in response to this multi-peptide DC vaccine.

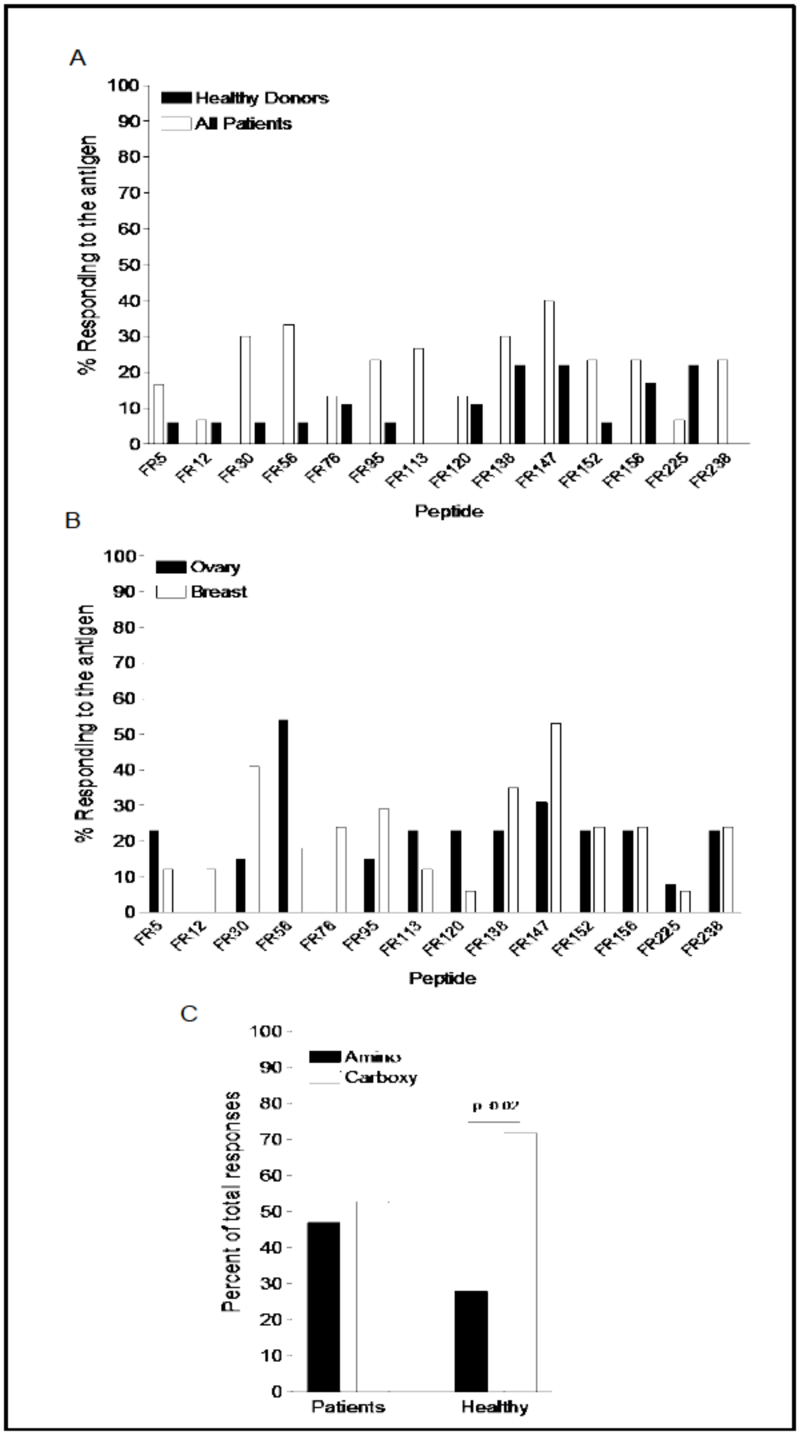

Fig 3. A high proportion of breast and ovarian cancer patients have T cell responses to folate receptor alpha (FR). (A) Proportions of each cohort responding to the individual epitopes. (B) Proportions of ovarian and breast cancer patients that responded to the individual peptides. (C) Distribution of responses among the amino or carboxy terminus halves of the FR.

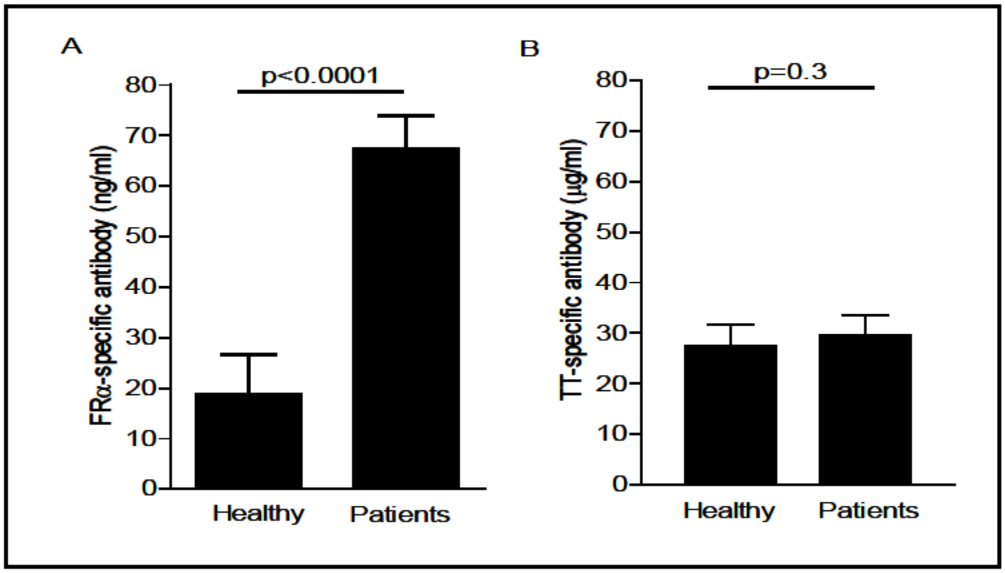

Fig 4. Patients with breast and ovarian cancer generate antibody responses to FRα. Panels A and B show the levels of antibody to the FR76 peptide and tetanus toxin, respectively. Each bar shows the mean (± s.e.m.) responses for both the patients (n=19) and healthy (n=11) volunteers. The p-values were calculated using a two-sided t test.

Table 1: FRα peptides to be used for FRα DC vaccine

SEQUENCE	POSITIONS	DESIGNATION	LENGTH (AA)
RTELLNVCMNAKHHKEK	30-46	FR30	17
QCRPWRKNACCSTNT	56-70	FR56	15
KDVSYLYRFNWNHCGEMA	76-93	FR76	18
LGPWIQQVDQSWRKERV	113-129	FR113	17
PWAAWPFLLSLALMLLWL	238-255	FR238	18

1.7 Investigational Agent

Th17-inducing DC loaded with folate receptor alpha (FRα) peptides

The investigational agent will be composed of autologous monocyte-derived DC loaded with Folate Receptor alpha (FRα) peptides. The DC will be prepared by culture of monocytes in GM-CSF and IL-4, followed by maturation with TNFα, IL-1β and PGE_2. DC will also be treated with IL-15 and a potent, selective, ATP-competitive and cell-permeable methylsulanylimidazole p38 MAP kinase inhibitor (Calbiochem Cat. No. 506121) throughout the manufacturing process. DC will be loaded with FRα peptides at the time of maturation.

The same set of FRα peptides (see Table 1, Section 1.5) are currently being evaluated in a Phase I trial of FRα peptide vaccination (Mayo Clinic protocol MC1015, ClinicalTrails.gov Identifier NCT01606241, IND #14546).

Dendritic cell activation of Th17 responses

This clinical trial is based on the premise that DC vaccination designed to drive a tumor antigen-specific Th17 T cell response holds the potential to be of clinical benefit for patients with ovarian cancer. DC are remarkable for their plasticity in directing T cell differentiation and effector function, and thus the key to success may reside in our ability to educate DC to drive ovarian tumor antigen-specific Th17 responses.

Several studies have indicated that regulation of the p38 and ERK MAPK signal transduction pathways in DC plays a central role in direction of T cell differentiation. Inhibition of MEK 1/2 and ERK MAPK signaling promotes IL-12 production and Th1 T cell responses, whereas inhibition of p38 MAPK increases signal transduction through ERK 1/2 and blocks IL-12 production [29]. At face value, these observations suggest that inhibition of p38 MAPK signaling would be disadvantageous for DC-driven anti-tumor T cell responses, since this would abrogate Th1 responses. However, p38 inhibition promotes differentiation and survival of monocyte-derived DC [30], and p38 inhibition or MEK/ERK MAPK activation restores deficiencies in DC function in myeloma patients [31], suggesting that treatment of DC with pharmacological inhibitors of p38 signaling may confer benefit. Of particular significance, blockade of the p38 pathway can attenuate regulatory T cell induction by DC [32], whereas blockade of the ERK pathway suppresses DC-driven Th17 responses [33], suggesting that p38 blockade (which enhances ERK phosphorylation) may favor a switch from Treg induction to Th17 differentiation and expansion.

Preclinical studies

Treatment of ovarian tumor antigen-loaded, cytokine-matured DC with a combination of IL-15 and a p38 MAPK inhibitor affords synergy in antagonism of Treg induction and redirection toward Th17 responses that correlate with strong $CD8^+$ CTL activation [34] (manuscript included in Section 8). Furthermore, DC vaccination of mice bearing advanced ovarian tumors has shown that ex vivo pharmacological inhibition of p38 signaling in vaccine DC yields an enhanced survival rate that correlates with increased Th17 frequencies.

O*utcomes of IL-15/p38 MAPK inhibitor treatment of human monocyte-derived DC, relative to cytokine-matured DC:*

a. Diminished activation and expansion of $Foxp3^+$ $CD4^+$ Treg and strong activation of tumor antigen-specific Th17 responses.
b. Potent activation of tumor antigen-specific $CD8^+$ CTL responses.
c. B7-H1 has been implicated in differentiation of adaptive $Foxp3^+$ Treg [8], and p38 MAPK inhibition leads to loss of B7-H1 expression by DC, suggesting a possible mechanism for reduced recruitment and activation of $CD4^+$ $Foxp3^+$ Treg.
d. Diminished $CD4^+$ T cell expression of CTLA-4, a co-inhibitory ligand associated with Treg function.

e. Diminished CD4^{+} T cell expression of PD-1, indicating the potential for reduced susceptibility to B7-H1 (PDL-1)-induced apoptosis or anergy in the tumor microenvironment.
f. Diminished DC expression of CD80 and CD86, but conserved expression of ICOS-L, suggesting a pattern of costimulation that favors Th17 responses [35].
g. IDO expression by DC contributes to Treg responses [9, 10]. IDO also inhibits Th17 responses [36], suggesting that IDO expression may play a key role in regulation of the Treg/Th17 balance. Inhibition of p38 signaling ablates IDO activity in DC.
h. Multiplex and flow cytometric analyses show increased ERK phosphorylation following p38 inhibition, suggesting that signal transduction via the ERK MAPK pathway in DC is associated with recruitment of Th17 responses.
i. In the ID8 mouse model of ovarian cancer, therapeutic vaccination with cytokine matured rAAV-SP17-transduced DC prolonged survival of tumor-bearing mice, but all animals succumbed to disease. In contrast, therapeutic vaccination with DC treated with a p38 MAPK inhibitor resulted in disease-free survival of >300 days in 19/20 vaccinated animals.

Collectively, these results support the proposal that treatment of DC with p38 MAPK inhibition plus IL-15 will drive ovarian tumor antigen-specific Th17 and CTL responses, and further suggest that this innovative approach may offer the potential for effective DC vaccination against ovarian cancer.

1.8 Clinical data to date

There are no available clinical research data to date on the investigational product.

1.9 Dose Rationale and Risk/Benefits

1.91 Dosage, dosage regimen and dosage period

FRα peptide-pulsed DC will be administered at a vaccine dose of 15 x 10^6 DC (allowable range as release criterion is 10-20 x 10^6). The vaccine volume will be 0.8 mL and will be administered intradermally in order to increase proximity of vaccination and local lymph node draining basins for stimulation of the immune response. Approximately 0.1 mL will be injected in a single site, with the total volume being divided into eight injections split between two areas. The same areas may be used for repeated vaccinations, although areas may be rotated from cycle to cycle. Alternatively, the vaccine dose may be administered via the 3M hollow Microneedle Transdermal system (hMTS). The entire vaccine dose is loaded into the hMTS device and the cells are subsequently delivered at one site. The useable injections sites are the same as the BD microinjection needle. Only individuals trained in the use of the 3M hMTS device can perform the injections.

Five DC vaccines will be administered at 21 day intervals, plus or minus 3 days to maximize patient convenience and protocol adherence. The dose and schedule is based on past experience of DC vaccine trials for gynecological malignancies [37].

Patients who tolerate treatment and do not have symptomatic recurrence of OC may continue therapy to include vaccination every 3 months from the completion of the 5th vaccination for a period of up to 2 years, or until progressive/recurrent disease is confirmed. Duration of potential extension, and the option for such treatment, will be

patient specific as supply of additional vaccinations varies based on quantity produced at study initiation.

1.92 Rationale for selection of dose

A phase I dose escalation clinical trial of DC vaccination in patients with cervical cancer indicated optimal stimulation of tumor antigen-specific T cell responses with a dose of 1.5×10^7 DCs in injection-grade saline containing 20% heat-inactivated autologous serum, delivered s.c. and intradermal (intradermal preferred) at 14 day intervals [37].

Leukapheresis (10 liter volume) followed by CliniMACS isolation of $CD14^+$ cells and high density DC culture in G-Rex flasks provided an optimal yield of 2.5×10^8 mature DC from 10^9 $CD14^+$ cells. This yield is sufficient for cryopreservation of 12 vials at 2×10^7 DC/vial, which is the maximum number of DC vaccine treatments under this protocol (see Section 5.3).

1.93 Potential risks and benefits

Known toxicities associated with DC vaccination:

- DC vaccination has not resulted in significant systemic toxicity in the clinical trials that have been performed. Of the adverse effects noted, mild symptoms, such as low-grade fever and local reactions at the injection site, were most common.
- In a recently conducted phase I clinical trial of DC vaccination of patients with early-stage cervical cancer (BB-IND 11307), no adverse side effects were observed or reported by subjects following immunization beyond the immediate discomfort associated with injection. We noticed, however, local reactions (mild erythema, swelling/induration, pruritus) at the subcutaneous vaccination sites that increased with the number of vaccinations in most of the patients. We also observed a slight enlargement in the draining lymph node in the groin after DC injections in some of the patients. The patients were monitored during treatment with complete blood counts and serum chemistries that included liver and renal function tests and electrolytes. No alterations in liver and renal function were detected.
- One of the major concerns regarding DC vaccination with self tumor antigens is the possible induction of autoimmunity. Vitiligo has been seen in some melanoma patients, but no cases of severe autoimmune reactions have been reported. The target antigen in this trial, FRα, is expressed at low levels in some normal tissues.
- The possible risks of stimulation of Th17 responses through DC vaccination in ovarian cancer patients are not known. Although a number of studies have documented potent anti-tumor activity for Th17 T cells, other studies have reported pro-angiogenic and tumor-promoting properties ascribed to Th17 immune responses [38, 39]. Th17 responses are pro-inflammatory, and may be associated with as yet unknown side effects. All subjects will be monitored closely for treatment-related adverse events and dose-limiting toxicities.

Known toxicities associated with FRα vaccination:

Vaccination with FRα peptides with GM-CSF as an adjuvant is currently being tested in the clinical protocol MC1015. Thus far, 18 patients have initiated treatment, and 7

patients have completed planned study treatment. One patient had grade 4 sepsis, which was attributed as unrelated to treatment. One patient had a grade 3 injection site reaction manifesting as an ulceration. Grade 2 adverse events are as shown in Table 2:

Table 2: Toxicities associated with FRα vaccination in the clinical protocol MC1015

Treatment	Number of patients	Grade 2+ toxicities (number of patients)
Cyclophosphamide	19 (1 off study before starting vaccine)	Grade 4: Sepsis (1)<br>Grade 3: (0)<br>Grade 2: Fatigue (1) , nausea (1), sinus pain (1), ↓ lymphocytes (3), ↓ neutrophils (2), ↓ WBC (3)
Vaccine	18 (1 cycle: 4, 3 cycles: 2, 4 cycles: 2, 5 cycles: 3, 6 cycles: 7)	Grade 4: (0)<br>Grade 3: Injection site reaction (1)<br>Grade 2: Fatigue (2), injection site reaction (1), dyspnea (1), arthralgia (1), myalgia (1), eye disorder (1), upper respiratory infection (1), ↓ neutrophils (2), ↓ WBC (1), headache (1), incoordination (1), upper respiratory infection (1), stomatitis (1)

2.0 Goals

2.1 Primary objective:

Determine the safety and tolerability of FRαDC vaccination.

2.2 Secondary objectives:

2.21 Measure time to disease recurrence of patients treated with FRαDCs.

2.22 Measure overall survival of patients treated with FRαDCs.

2.3 Correlative objectives:

2.31 Determine whether FRαDC vaccination induces an increase in the number of FRα-specific IL-17-secreting Th cells, as determined by ELISpot.

2.32 Determine whether FRαDC vaccination induces an increase in the number of FRα-specific T cells that secrete IFNγ, TNFα, IL-10, and Granzyme B, as determined by ELISpot.

2.33 Determine whether FRαDC vaccination induces antibodies specific for FRα.

2.34 Determine whether FRαDC vaccination induces a DTH skin reaction specific for FRα.

2.35 Measure FRα expression in patients' primary tumors and in tumors that recur after FRαDC vaccine treatment (when available).

2.36 Determine whether FRαDC vaccination is associated with changes in peripheral blood immune cell subsets.

2.37 Determine whether FRαDC vaccination leads to increases in plasma antibodies directed against OC-associated antigens, and whether changes in antibody levels are associated with recurrence-free survival.

3.0 Patient Eligibility

Prior to discussing protocol entry with the patient, call the MCCC Registration Office [redacted] to insure that a place on the protocol is open to the patient.

3.1 Inclusion Criteria

3.11 Age ≥18 years.

3.12 Histologically confirmed surgical diagnosis of stage IIIC or stage IV epithelial ovarian, fallopian tube, or primary peritoneal cancer. Patients with stage III cancer must have had peritoneal metastasis beyond pelvis more than 2 cm in greatest dimension and/or regional lymph node metastasis. NOTE: Histologic confirmation of the primary tumor is required. Eligible histologies include serous, endometrioid, clear cell, mucinous, transitional cell, undifferentiated, or mixed carcinoma.

3.13 Completion of cytoreductive surgery and has completed one (and only one) course of platinum-based chemotherapy (5-9 cycles) ≥ 4 but ≤20 weeks prior to registration.

NOTE: Cytoreductive surgery may have been prior to or after the first cycle of chemotherapy but must include hysterectomy and bilateral salpingo-oophorectomy, if the uterus and/or ovaries had not previously been removed.

NOTE: Patients may have had more than one chemotherapy regimen (ex: paclitaxel/ carboplatin switched to docetaxel/carboplatin due to allergy; weekly treatment switched to every 3 week treatment due to intolerance), but may not have received a separate course of treatment for recurrent OC.

NOTE: Patients may receive both neoadjuvant and adjuvant chemotherapy provided both regimens are platinum-based and total 9 or fewer chemotherapy cycles.

3.14 No evidence of disease at the time of registration, including no clinical concern for disease recurrence based on each of the following:

- No evidence of disease by history and physical exam
- CA125 within normal limits
- CT abdomen/pelvis demonstrating no radiological evidence of disease performed after completion of chemotherapy ≤28 days before entering study

3.15 ECOG performance status 0 or 1 (Appendix I).

3.16 The following laboratory values obtained ≤28 days prior to registration.

- Absolute neutrophil count (ANC) ≥1.0 x 10^9/L
- Platelet count ≥75 x 10^9/L
- Hemoglobin ≥8.5 g/dL
- Lymphocytes ≥0.3x 10^9/L
- Total bilirubin ≤2 x upper limit of normal (ULN), unless patient has a documented history of Gilbert's disease, then Direct bilirubin ≤1.0 mg/dL.
- Aspartate transaminase (AST) ≤3 x ULN
- Creatinine ≤2.0 mg/dL
- Monocytes ≥ 0.25 x 10^9/L

3.17 Able to provide informed written consent.

3.18 Expected survival >6 months.

3.19a Willingness to return to Mayo Clinic Rochester for follow-up appointments.

3.19b Willingness to provide blood samples for immune assessment and other tests (see Sections 4.0, 6.2, 14).

3.19c Willingness to undergo a tetanus vaccination.

3.2 Exclusion Criteria

3.21 Co-morbid systemic illnesses or other severe concurrent disease which, in the judgment of the investigator, would make the patient inappropriate for entry into this study or interfere significantly with the proper assessment of safety and toxicity of the prescribed regimens.

3.22 Immunocompromised patients and patients known to be HIV positive and currently receiving antiretroviral therapy.
NOTE: Patients known to be HIV positive, but without clinical evidence of an immunocompromised state, are eligible for this trial.

3.23 Uncontrolled intercurrent illness including, <u>but not limited to</u>:

- Ongoing or active infection
- Symptomatic congestive heart failure
- Unstable angina pectoris
- Cardiac arrhythmia
- Psychiatric illness/social situations that would limit compliance with study requirements
- Other uncontrolled intercurrent illness (specify)

3.24 Receiving any other investigational agent which would be considered as a treatment for the primary neoplasm.

3.25 Other active malignancy ≤3 years prior to registration.
EXCEPTIONS: Non-melanotic skin cancer or carcinoma-in-situ of the cervix.
NOTE: If there is a history or prior malignancy, they must not be receiving other specific treatment for their cancer.

3.26 History of myocardial infarction ≤6 months prior to registration, or congestive heart failure requiring use of ongoing maintenance therapy for life-threatening ventricular arrhythmias.

3.27 Epithelial ovarian cancer of low malignant potential (borderline tumor).

3.28 Treatment with chemotherapy, radiation therapy, or other immunotherapy ≤4 weeks prior to registration.

3.29a Immunosuppressive therapy (excluding topical steroids) for any other condition ≤4 weeks prior to registration.

3.29b Persistent fever (>24 hours) documented by repeated measurement ≤4 weeks prior to registration

3.29c Diagnosis of autoimmune disease, including, but not limited to:
- Systemic lupus erythematosus (lupus)
- Multiple sclerosis (MS)
- Rheumatoid arthritis (RA)
- Ankylosing spondylitis
- Other autoimmune disease (specify)

3.29d Use of a systemic steroid (>5 mg prednisone daily or equivalent) ≤4 weeks prior to registration.

4.0 Test Schedule

Tests and Procedures	Prior to Treatment		Active Treatment Phase			At the time of treatment discontinuation due to PD
	≤28 days prior to registration	Week 0	Prior to (≤3 days of) Cycles 1, 2, 3, and 5 (Weeks 4, 7, 10, and 16)	Prior to (≤3 days of) Cycles 4, 6, 7, 8, 9, 10, 11, and 12 (Weeks 13, 27, 39, 52, 65, 78, 91, and 104)	Three weeks after Cycles 5 and 12 (Weeks 19 and 107) ±14 days	
History, exam, weight, adverse event assessment, ECOG PS	X		X[R]	X	X[R]	X
Neurologic Evaluation[1]	X		X	X	X	X
Height	X					
Tetanus vaccine[2,R]	X					
CT abdomen/pelvis	X					X
Submit tumor to pathology[5, R]	X					X
Hematology group[3]	X		X[R]	X	X[R]	X
Chemistry group[4]	X		X[R]	X	X[R]	X
CA-125	X			X	X	X
Research blood tests[6, R]	X		X	X	X	X
Apheresis[7,R]		X				
Treatment with FRαDCs[R]			X	X		
DTH testing with unloaded DCs[8, R]			X	X		

1. Should include review of systems (ROS) and a screening neurological examination. Neurologic evaluation includes assessment for cranial nerves, cerebellar and neuromuscular as well as joint and skin exam. See Appendix III.
2. If no prior tetanus shot within 1 year of registration.
3. Includes CBC with differential: Hemoglobin, white blood cells, absolute neutrophil count, absolute monocyte count, absolute lymphocyte count, platelet count.
4. Includes creatinine, AST, alkaline phosphatase, and total bilirubin, direct bilirubin (only draw if total bilirubin is elevated).
5. Submit primary tumor for testing of folate receptor alpha expression is optional. Submission of a biopsy specimen at the time of recurrence is optional (See Section 17).
6. Volumes and tests conducted on research blood samples are detailed in Section 14.
7. Apheresis will be performed once. However, if the apheresis does not yield FRαDCs released by the Human Cell Therapy Laboratory for patient use, one additional apheresis run may be performed (optional if the patient prefers to continue with the study). If this exception occurs, the date of the second apheresis run will be considered week 0.
8. For immune monitoring. This testing should be administered on the same day as FRαDCs. Perform only with Cycle 1, Cycle 5, and Cycle 12 (Weeks 4, 16, and 104).

R. Research-funded

5.0 Grouping Factors: None.

6.0 Registration/Randomization Procedures

Prior to discussing protocol entry with the patient, call the MCCC Registration Office [redacted] to insure that a place on the protocol is open to the patient.

6.1 To register a patient, fax [redacted] a completed eligibility checklist to the Mayo Clinic Cancer Center (MCCC) Registration Office between 8 a.m. and 4:30 p.m. central time Monday through Friday.

6.2 Correlative Research

A mandatory correlative research component is part of this study, the patient will be automatically registered onto this component (see Sections 3.19b, 4.0, 14.1*)*.

An optional correlative research component is part of this study, there will be an option to select if the patient is to be registered onto this component (see Section 17).

- Patient has/has not given permission to give her tissue sample for research testing.

6.3 Documentation of IRB approval must be on file in the Registration Office before an investigator may register any patients.

In addition to submitting initial IRB approval documents, ongoing IRB approval documentation must be on file (no less than annually) at the Registration Office [redacted] If the necessary documentation is not submitted in advance of attempting patient registration, the registration will not be accepted and the patient may not be enrolled in the protocol until the situation is resolved.

When the study has been permanently closed to patient enrollment, submission of annual IRB approvals to the Registration Office is no longer necessary.

6.4 Prior to accepting the registration, registration application will verify the following:
- IRB approval at the registering institution
- Patient eligibility
- Existence of a signed consent form
- Existence of a signed authorization for use and disclosure of protected health information

6.5 At the time of registration, the following will be recorded:
- Patient has/has not given permission to store and use his/her sample(s) for future research of ovarian cancer at Mayo.
- Patient has/has not given permission to store and use his/her sample(s) for future research to learn, prevent, or treat other health problems.
- Patient has/has not given permission for MCCC to give his/her sample(s) to researchers at other institutions.

6.6 Treatment cannot begin prior to registration and must begin ≤14 days after registration. (Only apheresis must begin within 14 days of registration.)

6.7 Pretreatment tests/procedures (see Section 4.0) must be completed within the guidelines specified on the test schedule.

6.8 Treatment on this protocol must commence at a Mayo Clinic Rochester institution under the supervision of a medical oncologist.

7.0 Protocol Treatment

7.1 Treatment Schedule

Agent	Dose Level	Route	Day	Retreatment
FRαDCs	1.5 x 10^7 DCs in 0.8 mL	intradermal	1	every three weeks (Cycles 1-5); every three months (Cycles 6-12)
Unloaded DCs	1.5 x 10^7 DCs in 0.8 mL	intradermal	1	Cycles 1, 5, and 12 (Weeks 4, 16, and 104)

7.2 For this protocol, the patient must return to the treating institution for evaluation at least every three weeks (±3 days) during induction treatment (Cycles 1-5) and every 3 months (±14 days) during maintenance treatment (Cycles 6-12).

7.3 Dose limiting toxicity
For this protocol, a dose limiting toxicity is defined as any of the following adverse events observed during the first cycle of treatment that is considered to be definitely, probably, or possibly related to the study treatment as per NCI Common Terminology Criteria for Adverse Events.

**Adverse Event**	**DLT Definition***
Hematologic	ANC <0.5 x 10^9/L or PLT <50 x 10^9/L
Bone pain<br>Myalgia/arthralgia	≥Grade 3 and persisting >72 hours
Systemic hypersensitivity reactions/Allergic reactions (except injection site reactions)	≥Grade 2
Injection site reactions	≥Grade 3 and persisting >72 hours
Autoimmune disorders involving major organs (including autoimmune colitis, hemolysis, nephritis, vasculitis, or hepatitis)	≥Grade 2
Autoimmune disorders that do not involve major organs (e.g. arthritis, thyroiditis)	≥Grade 2 and persisting >2 weeks
All other non-hematologic involving major organs (including heart, lungs, liver, small bowel, colon, central nervous system, and kidneys)	≥Grade 3
All other non-hematologic not involving major organs	≥Grade 3 and persisting >72 hours
All categories (if possible, probable, or definite attribution to study treatment)	Grade 4

* Any adverse event that requires treatment discontinuation per Section 8.0 will be considered a DLT.

7.31 Treatment by a local medical doctor is not allowed.

7.32 Three patients will be treated with cycle 1 of the vaccine and observed for a minimum of 21 days, to assess toxicities, before new patients are treated.

7.321 If zero or one of the first three patients experiences DLT, the study will open to accrual for three additional patients.

7.323 If two or three of the first three patients experiences DLT, the study will be temporarily closed until the data are reviewed by the study investigators and the Data Safety Monitoring Board.

7.33 Three additional patients will be treated with cycle 1 of the vaccine and observed for a minimum of 21 days, to assess toxicities, before new patients are treated.

7.321 If zero or one of the first six patients experiences DLT, the study will open to accrual for all remaining patients.

7.322 If two or more of the first six patients experiences DLT, the study will be temporarily closed until the data are reviewed by the study investigators and the Data Safety Monitoring Board.

7.34 Investigators are to contact the Study Chair as soon as any dose-limiting toxicity (DLT) occurs.

8.0 Dosage Modification Based on Adverse Events

ADR reporting may be required for some adverse events (See Section 10)

→ → Use the NCI Common Terminology Criteria for Adverse Events (CTCAE) version 4.0 unless otherwise specified ← ←

CTCAE System/Organ/Class (SOC)	ADVERSE EVENT	ACTION
BASED ON INTERVAL ADVERSE EVENT		
Investigations	Grade 3 neutrophil count (ANC <1.0 x 10^9/L)	Hold treatment until ANC ≥1.0 x 10^9/L (maximum 2 weeks)
	Grade 4 neutrophil count (ANC <0.5 x 10^9/L)	Discontinue treatment and go to Event Monitoring
	Grade 2 platelet count (decreased <75 x 10^9/L)	Hold treatment until PLT ≥75 x 10^9/L (maximum 2 weeks)
	Grade ≥3 platelet count (decreased <50 x 10^9/L)	Discontinue treatment and go to Event Monitoring
Musculoskeletal disorders	Grade ≥2 bone pain	Hold treatment until ≤Grade 1 if possible, probable, or definite attribution to study treatment*
	Grade 2 bone pain persisting >4 weeks or grade ≥3 bone pain persisting >72 hours	Discontinue treatment and go to Event Monitoring if possible, probable, or definite attribution to study treatment*
	Grade ≥2 arthralgia	Hold treatment until ≤Grade 1 if possible, probable, or definite attribution to study treatment*
	Grade 2 arthralgia persisting > 4 weeks or grade ≥3 arthralgia persisting >72 hours	Discontinue treatment and go to Event Monitoring if possible, probable, or definite attribution to study treatment*
	Grade ≥ 2 myalgia	Hold treatment until ≤Grade 1 if possible, probable, or definite attribution to study treatment*

CTCAE System/Organ/Class (SOC)	ADVERSE EVENT	ACTION
	Grade 2 myalgia persisting >4 weeks or Grade ≥3 myalgia persisting >72 hours	Discontinue treatment and go to Event Monitoring if possible, probable, or definite attribution to study treatment*
Skin and subcutaneous tissue disorders	Grade 2 rash	Hold treatment until ≤Grade 1 if possible, probable, or definite attribution to study treatment*
	Grade 2 rash persisting >4 weeks or ≥Grade 3 rash persisting >72 hours	Discontinue treatment and go to Event Monitoring if possible, probable, or definite attribution to study treatment*
Immune system disorders	Grade ≥2 allergic reaction (except injection site reactions)	Discontinue treatment and go to Event Monitoring
	Grade 2 allergic reaction (Injection site reaction)	Hold treatment until ≤Grade 1 (maximum 4 weeks)
	Grade ≥3 allergic reaction (Injection site reaction) persisting >72 hours	Discontinue treatment and go to Event Monitoring
	Grade ≥2 autoimmune disorder involving major organs (including autoimmune colitis, hemolysis, nephritis, vasculitis, or hepatitis)	Discontinue treatment and go to Event Monitoring
	Grade ≥2 autoimmune disorders that do not involve major organs (e.g. arthritis, thyroiditis) persisting >2 weeks	Discontinue treatment and go to Event Monitoring
Other	Grade ≥3 Non-hematologic involving major organs (including heart, lungs, liver, small bowel, colon, central nervous system, and kidneys)	Discontinue treatment and go to Event Monitoring
	Grade ≥3 non-hematologic not involving major organs but persisting >72 hours	Discontinue treatment and go to Event Monitoring
All	Grade 4	Discontinue treatment and go to Event Monitoring if possible, probable, or definite attribution to study treatment*

*Musculoskeletal disorders and skin and subcutaneous disorders that do not have an obvious cause unrelated to study treatment (eg. trauma, sunburn) should be considered at least possible attribution to the study treatment, and the appropriate action should be taken.

9.0 Ancillary Treatment/Supportive Care

9.1 Antiemetics

Antiemetics may be used at the discretion of the attending physician.

9.2 Full supportive care

Patients should receive full supportive care while on this study. This includes blood product support, antibiotic treatment, and treatment of other newly diagnosed or concurrent medical conditions. All blood products and concomitant medications such as antidiarrheals, analgesics, and/or antiemetics received from the first day of study treatment administration until 30 days after the final dose will be recorded in the medical records.

9.3 Acute vaccine reactions

9.31 Fever: Fever may be treated symptomatically with acetaminophen 650-1000 mg by mouth every six hours as needed. Maximum total daily dose of acetaminophen is 4000 mg/24 hrs.

9.32 Myalgias/arthralgias: Myalgias and arthralgias may be treated symptomatically with acetaminophen 650-1000 mg by mouth every six hours as needed.

9.33 Injection site reactions: Erythema and induration are common injection site reactions that are expected to be self-limited. Injection site reactions should not be treated unless causing significant symptoms. If intense pruritis occurs, this should be treated with over-the-counter topical or antihistamines. If topical antihistamines are ineffective, over-the-counter oral anti-histamines and/or topical steroids may be used.

9.4 Immune-related adverse events

Immune-related adverse events are toxicities associated with vaccine treatment for which the pathophysiology is consistent with an immune mechanism. If dose-limiting immune-related adverse events occur, these should be treated with prednisone 1 mg/kg, given once daily or in two divided doses per the discretion of the treating investigator. Prednisone should continue at the starting dose for at least two weeks and until symptoms improve to grade 1 or lower. At that point, prednisone may be tapered per the discretion of the treating investigator.

9.5 No ice on injection site

Ice should not be used on injection sites within 24 hours of vaccine administration.

10.0 Adverse Event (AE) Reporting and Monitoring

10.1 Adverse Event Characteristics

CTCAE term (AE description) and grade: The descriptions and grading scales found in the revised NCI Common Terminology Criteria for Adverse Events (CTCAE) version 4.0 will be utilized for AE reporting. All appropriate treatment areas should have access to a copy of the CTCAE version 4.0. A copy of the CTCAE version 4.0 can be downloaded from the CTEP web site: (http://ctep.cancer.gov/protocolDevelopment/electronic_applications/ctc.htm)

10.11 Adverse event monitoring and reporting is a routine part of every clinical trial. First, identify and grade the severity of the event using the CTCAE version 4.0. Next, determine whether the event is expected or unexpected (see Section 10.2) and if the adverse event is related to the medical treatment or procedure (see Section 10.5). With this information, determine whether the event must be reported as an expedited report (see Section 10.). Expedited reports are to be completed within the timeframes and via the mechanisms specified in Sections 10.4. All AEs reported via expedited mechanisms must also be reported via the routine data reporting mechanisms defined by the protocol (see Sections 10.6 and 18.0).

10.12 Each CTCAE term in the current version is a unique representation of a specific event used for medical documentation and scientific analysis and is a single MedDRA Lowest Level Term (LLT). Grade is an essential element of the Guidelines and, in general, relates to **severity** for the purposes of regulatory reporting to NCI.

NOTE: A severe AE, as defined by the above grading scale, is **NOT** the same as serious AE which is defined in the table in Section 10.4.

10.2 Expected vs. Unexpected Events

- The determination of whether an AE is expected is based on agent-specific information provided in Section 15.0 of the protocol and the study specific consent form.
- Unexpected AEs are those not listed in the agent-specific information provided in Section 15.0 of the protocol and the study specific consent form.

NOTE: "Unexpected adverse experiences" means any adverse experience that is neither identified in nature, severity, or frequency of risk in the information provided for IRB review nor mentioned in the consent form.

10.3 Assessment of Attribution

When assessing whether an adverse event is related to a medical treatment or procedure, the following attribution categories are utilized:

Definite - The adverse event *is clearly related* to the agent(s).
Probable - The adverse event *is likely related* to the agent(s).
Possible - The adverse event *may be related* to the agent(s).
Unlikely - The adverse event *is doubtfully related* to the agent(s).
Unrelated - The adverse event *is clearly NOT related* to the agent(s).

Events determined to be possibly, probably or definitely attributed to a medical treatment suggest there is evidence to indicate a causal relationship between the drug and the adverse event.

10.4 Expedited Reporting Requirements for IND/IDE Agents

Phase 1 and Early Phase 2 Studies: Expedited Reporting Requirements for Adverse Events that Occur on Studies under an IND/IDE within 30 Days of the Last Administration of the Investigational Agent/Intervention [1, 2]

FDA REPORTING REQUIREMENTS FOR SERIOUS ADVERSE EVENTS (21 CFR Part 312)

NOTE: Investigators **MUST** immediately report to the sponsor **ANY** Serious Adverse Events, whether or not they are considered related to the investigational agent(s)/intervention (21 CFR 312.64)

An adverse event is considered serious if it results in **ANY** of the following outcomes:

1) Death
2) A life-threatening adverse event
3) An adverse event that results in inpatient hospitalization or prolongation of existing hospitalization for ≥ 24 hours
4) A persistent or significant incapacity or substantial disruption of the ability to conduct normal life functions
5) A congenital anomaly/birth defect.
6) Important Medical Events (IME) that may not result in death, be life threatening, or require hospitalization may be considered serious when, based upon medical judgment, they may jeopardize the patient or subject and may require medical or surgical intervention to prevent one of the outcomes listed in this definition. (FDA, 21 CFR 312.32; ICH E2A and ICH E6).

ALL SERIOUS adverse events that meet the above criteria MUST be immediately reported to the sponsor within the timeframes detailed in the table below.

Hospitalization	Grade 1 and Grade 2 Timeframes	Grade 3-5 Timeframes
Resulting in Hospitalization ≥24 hrs	7 Calendar Days	24-Hour 3 Calendar Days
Not resulting in Hospitalization ≥24 hrs	Not required	

Expedited AE reporting timelines are defined as:

- "24-Hour; 3 Calendar Days" - The AE must initially be reported within 24 hours of learning of the AE, followed by a complete expedited report within 3 calendar days of the initial 24-hour report.
- "7 Calendar Days" - A complete expedited report on the AE must be submitted within 7 calendar days of learning of the AE.

[1] Serious adverse events that occur more than 30 days after the last administration of investigational agent/intervention and have an attribution of possible, probable, or definite require reporting as follows:

Expedited 24-hour notification followed by complete report within 3 calendar days for:

- All Grade 3, 4, and Grade 5 AEs

Expedited 7 calendar day reports for:

- Grade 2 AEs resulting in hospitalization or prolongation of hospitalization

[2] For studies using PET or SPECT IND agents, the AE reporting period is limited to 10 radioactive half-lives, rounded UP to the nearest whole day, after the agent/intervention was last administered. Footnote "1" above applies after this reporting period.

Effective Date: May 5, 2011

Additional Instructions:

1. An increased incidence of an expected adverse event (AE) is based on the patients treated for this study at their site. A list of known/expected AEs is reported in the package insert or the literature, including AEs resulting from a drug overdose.
2. Submit MedWatch form 3500A to the FDA, MedWatch, 5600 Fishers Lane, Rockville, MD 20852-9787, by fax at 1-800-332-0178 or online at http://www.fda.gov/Safety/MedWatch/HowToReport/default.htm.

Mayo Clinic Cancer Center (MCCC) Institutions: Complete MCCC SAE Reporting Form online at http://livecycle2.mayo.edu/workspace/?startEndpoint=MC4158-56/Processes/MC4158-56-Process.MC4158-56 and attach copies of the MedWatch report. This process will automatically forward the information to the MCCC Regulatory Affairs Unit (RAU) Risk Information Specialist who will determine and complete IRB reporting. The RAU will submit to the MCCC SAE Coordinator and the MCCC IND Coordinator to determine if FDA submission is needed.

10.5 Other Required Reporting

10.51 Persistent or Significant Disabilities/Incapacities

Any AE that results in persistent or significant incapacity or substantial disruption of the ability to conduct normal life functions (formerly referred to as disabilities), congenital abnormities or birth defects, must be reported immediately if they occur at any time following treatment with an agent under an IND/IDE since they are considered to be a serious AE and must be reported to the sponsor as specified in 21 CFR 312.64(b).

10.52 Death

Any death occurring within 30 days of the last dose, regardless of attribution to an agent/intervention under an IND/IDE requires expedited reporting within 24-hours.

Any death occurring greater than 30 days with an attribution of possible, probable, or definite to an agent/intervention under an IND/IDE requires expedited reporting within 24-hours.

Reportable categories of Death

- Death attributable to a CTCAE term.
- Death Neonatal: A disorder characterized by cessation of life during the first 28 days of life.
- Death NOS: A cessation of life that cannot be attributed to a CTCAE term associated with Grade 5.
- Sudden death NOS: A sudden (defined as instant or within one hour of the onset of symptoms) or an unobserved cessation of life that cannot be attributed to a CTCAE term associated with Grade 5.
- Death due to progressive disease should be reported as **Grade 5 "Neoplasms benign, malignant and unspecified (including cysts and polyps) – Other (Progressive Disease)"** under the system organ class (SOC) of the same name. Evidence that the death was a manifestation of underlying disease (e.g., radiological changes suggesting tumor growth or progression: clinical deterioration associated with a disease process) should be submitted.

10.53 Secondary Malignancy

- A ***secondary malignancy*** is a cancer caused by treatment for a previous malignancy (e.g., treatment with investigational agent/intervention, radiation or chemotherapy). A secondary malignancy is not considered a metastasis of the initial neoplasm.
- All secondary malignancies that occur following treatment with an agent under an IND/IDE be reported. Three options are available to describe the event:
    - Leukemia secondary to oncology chemotherapy (e.g., Acute Myelocytic Leukemia [AML])

- Myelodysplastic syndrome (MDS)
- Treatment-related secondary malignancy

- Any malignancy possibly related to cancer treatment (including AML/MDS) should also be reported via the routine reporting mechanisms outlined in each protocol.

10.54 Second Malignancy

- A second malignancy is one unrelated to the treatment of a prior malignancy (and is NOT a metastasis from the initial malignancy). Second malignancies require ONLY routine reporting.

10.6 Required Routine Reporting

Adverse events to be graded at each evaluation and pretreatment symptoms/conditions to be evaluated at baseline per the CTCAE v4.0 grading unless otherwise stated in the table below:

**System Organ Class (SOC)**	**Adverse event/Symptoms**	**Baseline**	**Each evaluation**
Investigations	Neutrophil count decreased	x	x
	Platelet count decreased	x	x
Musculoskeletal and connective tissue disorders	Bone pain	x	x
	Arthralgia	x	x
	Myalgia	x	x
Skin and subcutaneous tissue disorders	Rash maculopapular	x	x
Immune system disorders	Allergic reaction	x	x
	Autoimmune disorder	x	x
General disorders and administration site conditions	Injection site reaction	x	x

10.61 Submit via appropriate MCCC Case Report Forms (i.e., paper or electronic, as applicable) the following AEs experienced by a patient and not specified in Section 10.6:

10.611 Grade 1 and 2 AEs deemed *possibly, probably, or definitely* related to the study treatment or procedure.

10.612 Grade 3 and 4 AEs regardless of attribution to the study treatment or procedure.

10.613 Grade 5 AEs (Deaths)

10.6131 Any death within 30 days of the patient's last study treatment or procedure regardless of attribution to the study treatment or procedure.

10.6132 Any death more than 30 days after the patient's last study treatment or procedure that is felt to be at least possibly treatment related must also be submitted as a Grade 5 AE, with a CTCAE type and attribution assigned.

11.0 Treatment Evaluation

11.1 For the purposes of this study, patients should be re-evaluated for tumor recurrence with physical exam and laboratory testing every three months, including CA-125.

11.11 Imaging evaluation will take place at the discretion of the treating investigator if there is clinical or laboratory suspicion for ovarian cancer recurrence, including concerning clinical findings or increase in CA-125 >35 U/ml.

11.12 If a patient has a CA-125 >35 U/ml, then a second CA-125 should be obtained at least three weeks later.

11.2 At the time of reevaluation, patients will be classified in the following manner:

11.21 No evidence of disease (NED): Patients will be considered to have NED if both of the following conditions are met:

11.211 The patient has a CA-125 ≤35 U/mL and has no symptom that the treating investigator suspects to be due to disease recurrence. (If a single CA-125 level is >35 U/mL, the patient is still considered NED until a confirmatory CA-125 at least 3 weeks after the initial elevated CA-125 is also >35 U/mL.)

11.212 There is no evidence of disease on any imaging tests performed. (Imaging is not required unless deemed clinically necessary by the treating investigator.)

11.22 Recurrent disease (PD): Patients will be considered to have PD if any of the following conditions are met:

11.221 The patient has a CA-125 >35 U/mL that has been confirmed by a second elevated CA-125 at least 3 weeks later.

11.222 There is evidence of disease on any imaging test performed.

12.0 Descriptive Factors

12.1 AJCC Stage: IIIC vs. IV

12.2 Debulking status: No gross residual disease vs. residual disease

13.0 Treatment/Follow–up Decision at Evaluation of Patient

13.1 Patients who are NED will continue treatment per protocol.

13.2 Patients who develop symptomatic PD while receiving therapy will go to the event-monitoring phase.

13.3 Patients who develop PD but do not have any symptoms that the treating investigator believes are caused by EOC may continue treatment per protocol for as long as they remain asymptomatic and the treating investigator and patient believe there is benefit to continuing therapy.

13.4 Patients who go off protocol treatment for reasons other than PD will go to the event-monitoring phase per Section 18.0.

13.5 If a patient fails to complete the first cycle of treatment for reasons other than toxicity, the patient will be regarded as non-evaluable and will be replaced.

13.6 A patient is deemed *ineligible* if after registration, it is determined that at the time of registration, the patient did not satisfy each and every eligibility criteria for study entry. The patient will go directly to the event-monitoring phase of the study (or off study, if applicable).

- If the patient received treatment, all data up until the point of confirmation of ineligibility must be submitted. Event monitoring will be required per Section 18.0 of the protocol.
- If the patient never received treatment, on-study material must be submitted. Event monitoring will be required per Section 18.0 of the protocol.

13.7 A patient is deemed a *major violation*, if protocol requirements regarding treatment in cycle 1 of the initial therapy are severely violated that evaluability for primary end point is questionable. All data up until the point of confirmation of a major violation must be submitted. The patient will go directly to the event-monitoring phase of the study. The patient may continue treatment off-protocol at the discretion of the physician as long as there are no safety concerns, and the patient was properly registered. Event monitoring will be required per Section 18.0 of the protocol.

13.8 A patient is deemed a *cancel* if he/she is removed from the study for any reason before any study treatment is given. On-study material and the End of Active Treatment/Cancel Notification Form must be submitted. No further data submission is necessary.

14.0 Body Fluid Biospecimens

14.1 Summary table of research blood and body fluid specimens to be collected for this protocol

Assessment	Blood or Body Fluid being Collected	Type of Collection Tube (color of tube top)	Volume to collect per tube	Prior to registration (mandatory)	Prior to (≤3 days before) each treatment cycle (mandatory)	At the time of treatment discontinuation due to PD (mandatory)	Three weeks after Cycles 5 and 12 (Weeks 19 and 107) (mandatory)	Temperature Conditions for Storage/ Shipping
Correlative immune tests	Blood	No Additive (red top)	5-20 mL[1]	20 mL (two 10 mL red top tubes)	5 mL (one 5 mL red top tube)	20 mL (two 10 mL red top tubes)	20 mL (two 10 mL red top tubes)	Ambient
Correlative immune tests	Blood	Sodium Heparin (green top)	20-180 mL[1]	180 mL (eighteen 10 mL green tops or three 60 mL heparin syringes)	20 mL (two 10 mL green top tubes)	180 mL (eighteen 10 mL green tops or three 60 mL heparin syringes)	180 mL (eighteen 10 mL green tops or three 60 mL heparin syringes)	Ambient

1. See Section 14.2 for detailed description of volumes

14.2 Collection and Processing

14.21 Blood collection

14.211 Prior to registration:

Collect 20 mL with no additive (two 10 mL red top tubes) and 180 mL with sodium heparin (eighteen 10 mL green top tubes or three 60 mL heparin syringes).

14.212 Prior to each cycle of treatment:

Collect 5 mL with no additive (one 5 mL red top tubes) and 20 mL with sodium heparin (two 10 mL green top tubes).

14.213 Three weeks after Cycles 5 and 12:

Collect 20 mL with no additive (two 10 mL red top tubes) and 180 mL with sodium heparin (eighteen 10 mL green top tubes or three 60 mL heparin syringes).

14.214 At the time of disease recurrence due to PD (mandatory):

Collect 20 mL with no additive (two 10 mL red top tubes) and 180 mL with sodium heparin (eighteen 10 mL green top tubes or three 60 mL heparin syringes).

14.22 Blood processing

14.221 No additive whole blood (red top tubes)

No mixing is required, but keep in upright position during transport.

14.222 Sodium heparin blood (green top tubes or sodium heparin syringes) Immediately mix at least 10 times by gentle inversion.

14.3 Shipping and handling

14.31 Kits will not be used for this study.

14.32 Send blood samples to Guggenheim 3-23, Mayo Clinic Rochester, Attn: Courtney Erskine via Mayo General Service.

14.4 Background and methodology

14.41 ELISpot Assays (enzyme-linked immunosorbent spot assays)

A 2-day ELISpot, which detects both activated and memory T cell effectors will be used to detect immunity to FRα as previously described [28, 40] (manuscript for reference 28 included in Section 8). Furthermore, the ELIspot assay is able to measure the frequency of the responding T cells per unit of peripheral blood mononuclear cells (PBMCs). On day 1, 3 x10^5 PBMCs/well will be plated into 96-well plates in 3-well replicates in 200 μl of RPMI-1640 containing L-glutamine, penicillin, streptomycin, and 10% fetal calf serum (T-cell medium) in the presence or absence of 10 mcg/ml peptide antigen, 1 mcg/ml protein, or 1 mcg/ml tetanus vaccine. The cells will be incubated at 37°C for 42-46 hours followed by washing three times with phosphate-buffered saline (PBS) containing 0.05% Tween-20. The plate will be incubated for 2 hours at 37°C in PBS with 5 mcg/ml biotinylated anti–cytokine Ab, washed in PBS, and further incubated with 100 mcl/well avidin-horseradish peroxidase (HRP, Vector Laboratories, Burlingame, CA) for 1 hour at room temperature. The anti-cytokine and biotinylated anti-cytokine antibody pair will be obtained from Mabtech (Sweden). After 3 washes in PBS, the plate will be incubated with 100 mcl/well HRP-colorimetric substrate (Vector Laboratories) for 5-20 minutes, rinsed with cool tap water, and allowed to dry completely. The nitrocellulose plates will be read on an AID ELIspot reader (Cell Technology, Inc., Columbia MD, reader software v.3.1.1.). A positive response is defined as a frequency that is significantly ($p < 0.05$, two-tailed t test) greater than the mean of control no-antigen wells and detectable (i.e., >1:100,000). A15-amino acid HLA-DR binding irrelevant peptide from Cyclin D1 will be used as a negative control peptide. Phytohemagglutinin (PHA) will be used as a positive control. The CEF viral peptide pool from the NIH AIDS Research and Reference Reagent Program

will be used to evaluate non-specific increases in immunity [28]. The CEF Control Peptide Pool is a group of 32 peptides, 8-12 amino acids in length, with sequences derived from the human Cytomegalovirus, Epstein-Barr Virus and Influenza Virus. A vaccine-induced increase in FRα-specific T cell responses will be defined as (1) a 2-fold or greater increase in FRα-specific T cells at any point during treatment if there were detectable pre-treatment levels of FRα-specific T cells or (2) FRα-specific T cells at any point during treatment if pre-treatment levels of FRα-specific T cells are non-detectable.

14.42 ELISAs (enzyme-linked immunosorbent assays)

Antibodies have made outstanding surrogates for response to vaccine. Furthermore, the FRα vaccine in this protocol incorporates a known antibody recognition epitope of FRα. Antigen (10μg/well), peptide or whole protein (tetanus protein will be included as a control) will be prepared in 0.06M carbonate buffer and added to ELISA microtiter plates for 24 hours. Plates will be washed with PBS and blocked with 3% BSA-PBS. One hundred microliters of diluted sera (1:125 for peptide and 1:40 for tetanus toxoid in 1%BSA-PBS) will be added and the plates further incubated for 2 hr at RT followed by washing with PBS/0.1% Tween-20. A 1:2000 dilution of anti-IgG-HRP is then added to wells for 1 hour followed by washing and color development after adding 100 μl TMB (3,3',5,5' tetramethylbenzidine) substrate to the wells. Color development is stopped with 50 μl of a 0.1N HCl solution. For the standard curve, serial dilutions of human IgG will be added to separate wells. As a peptide control, a peptide derived from human collagen II, HII.71 (PPGLTGPAGEPGRQGSPGAD), will be used. Optical densities will be read on a Victor V multiplate reader and concentrations of antibody will be determined using the standard curve with Graphpad Prism (Graphpad Software). A vaccine-induced increase in FRα-specific antibody responses will be defined as (1) a 2-fold or greater increase in FRα-specific antibody at any point during treatment if there were detectable pre-treatment levels of FRα-specific T cells or (2) FRα-specific antibodies at any point during treatment if pre-treatment levels of FRα-specific antibodies are non-detectable.

14.43 Peripheral blood immune cell subtype and ancillary studies

Cancer patients have well-defined alterations in circulating immune cells and are characterized by systemic reductions in T cell numbers and functions as well as increases in inhibitory cells like immunosuppressive monocytes and regulatory T cells. These systemic changes in patient immunity have significant implications for response to immunotherapy. Therefore, we will perform peripheral blood immunophenotyping using flow cytometry on patient samples. We will use a ten color 8 tube flow cytometry panel that encompasses lymphocyte, monocyte, granulocyte subsets, regulatory T cells, immunosuppressive monocytes and myeloid derived suppressor cells. This analysis will generate a detailed picture of the patient's peripheral blood immunophenotype and how they respond to the vaccine. We will also collect plasma at selected collection points that may be used to measure changes in cytokines and growth factors. We may also collect and store immune cell subtypes (such as T cells and monocytes) for transcriptome analysis or other analyses (DNA sequencing) to identify changes in the behavior of the immune response during the course of therapy.

14.44 RNASeq studies

Ovarian cancer patients expressing Th17 associated cytokines in the tumor microenvironment have a better prognosis than do patients not expressing this immune response signature. Why some patients achieve a Th17 protective response profile, while others do not, is unknown. We will examine the basis for these individual differences in immune responsiveness by studying peripheral blood cells collected in the planned blood draws. For the purpose of this study, we will analyze CD4+, CD8+ (primarily T cells), and CD14+ (primarily monocytes) lineage cells from 30 ml of freshly drawn peripheral blood. We will purify RNA from CD4, CD8, and CD14 cells and quantify gene expression by RNAseq. Samples will be collected at up to three time points, 1 prior to vaccination, 1 after completion of induction therapy, and 1 after treatment is completed or when the patients' disease progresses and they transition off protocol. Patient outcomes, including Th17 polarized responses to vaccination and time to disease progression and survival will be used to identify outcome-associated gene expression profiles. The results of this initial study will provide the framework for devising testable hypotheses by establishing outcome frequencies and associated gene expression profiles.

14.45 OC-associated autoantibody array

Antibodies against OC-associated antigens frequently arise during the course of OC and can be associated with clinical outcomes,[1] but the role played by anti-OC antibodies in the host immune response to OC is unclear. Patient plasma samples obtained prior to and at multiple time points after FRαDC vaccination will be added to high-density programmable protein microarrays containing multiple OC-associated antigens. Titers of pre- and post-vaccination autoantibodies will be compared, and changes in autoantibody titers will be correlated with recurrence-free survival. Samples will be sent to the laboratory of Karen S Anderson MD PhD who is jointly appointed at Mayo Clinic in Arizona and Arizona State University.

Attn: Marika Hopper
Biodesign Institute at Arizona State University
Center for Personalized Diagnostic – Anderson Lab A223
1001. S McAllister Dr
Tempe, AZ 85287
[redacted]

[1] Autoantibody signature for the serologic detection of **ovarian cancer**..
Anderson KS, Cramer DW, Sibani S, Wallstrom G, Wong J, Park J, Qiu J, Vitonis A, LaBaer J. J Proteome Res. 2015 Jan 2;14(1):578-86. doi: 10.1021/pr500908n. Epub 2014 Nov 17. PMID: 25365139

15.0 Drug Information

15.1 FRαDCs

IND number 15867

15.11 **Background**: The drug used in this study is patient's autologous dendritic cells (DC). Dendritic cells are cells manufactured in the laboratory from monocytes removed from patients' blood by leukapheresis. The dendritic cells need to acquire tumor antigens in order to stimulate anti-tumor immunity. Patients will be treated with DC exposed to peptides (small protein fragments) from the ovarian cancer-associated protein folate receptor alpha (FRα). DCs loaded with FRα peptides are referred to in this protocol as FRα DCs.

15.12 **Formulation**: The drug is supplied as recently thawed cells prepared from patient material. Cells are manufactured and released by the Human Cellular Therapy Lab, Mayo Clinic Rochester. Before being released for use, cells will undergo testing for sterility and potency.

15.13 **Preparation and storage**: Vaccine vials should be stored at less than -150ºC. The drug will be prepared and stored at the Human Cellular Therapy Lab, Mayo Clinic Rochester according to approved SOPs included in the IND. The drug will be delivered in a cooler on wet ice to the appropriate administration area by personnel from the Human Cell Therapy Lab.

15.14 **Administration:**

The vaccine dose may be administered via the 3M hollow Microneedle Transdermal system (hMTS). The entire vaccine dose is loaded into the hMTS device and the cells are subsequently delivered at one site. The useable injections sites are the same as the BD microinjection needle. Only individuals trained in the use of the 3M hMTS device can perform the injections.
Note: 3M hMTS needles are the first choice for delivery; the standard injection method will be used only if 3M hMTS needles or appropriately trained staff are not available.

15.141 Number and location of intradermal FRαDC doses
Each FRαDC dose is administered as 8 intradermal injections. For each injection, the total volume of the vaccine (0.8 mL) is evenly distributed among 8 injections. The FRαDC vaccine is injected into 4 intradermal sites premarked at least 3 cm apart from each other, at each of two areas of the body. The two areas of the body should be on the same side (left or right) in a given cycle, but should alternate between cycles. Approximately 0.1 mL of the vaccine will be injected at each site. Usable injection areas include: proximal upper extremities (deltoid regions), proximal lower extremities (anterior or lateral thigh regions at least 5 cm beyond hip and knee joints), left and right lower quadrants of the abdomen (at least 5 cm beyond inguinal region). The vaccine is administered using the Becton Dickinson intradermal (ID) needle, designed for multiple injections into the dermis.

In the induction phase, the interval between FRαDC doses is 3 weeks ±3 days. In the maintenance phase, the interval between doses is 3 months ±1 week.

15.142 Number and location of intradermal unloaded DC doses (for DTH testing)

On Cycles 1, 5, and 12, patients will receive both FRαDCs and unloaded DCs, which serve as a control for DTH skin testing. Each unloaded DC dose will be administered in the same total volume (0.8 mL) as the FRαDC dose, and should be administered in 8 injections. The unloaded DCs are injected into 4 intradermal sites premarked at least 3 cm apart from each other, at each of two areas of the body. These should be the same 2 areas of the body that the FRαDC vaccine is given for that cycle, but on the contralateral side of the body.

15.143 Intradermal Injection using the BD microneedle

- Only individuals trained in the use of the BD microneedle can perform the injections.
- Clean the skin with alcohol and allow it to dry prior to injection.
- Remove white cap from ID needle.
- Holding the syringe in your dominant hand, hold the skin taut with your other hand.
- Insert the needle perpendicular to the skin, in a short and quick movement.
- Maintain a light pressure on the syringe and inject slowly pushing the plunger with your index finger.
- Following injection, keep the needle inserted, remove the index finger, and wait a few seconds to prevent oozing.
- Using the same needle repeat injections for each site. The syringe plunger rod will be marked with guidelines for each injection
- Remove the needle from the skin and dispose the syringe into a sharps container.
- Document administration of the product on the Injection Site Record form (see appendix II).

15.144 Intradermal injection using the 3M hMTS device.

1. Select the injection site area and remove hair by shaving if necessary. Clean the area with alcohol and let dry. The injection site area should be free from skin pigmentation, skin damage, and scars.
2. Open the cover of the clamshell package and remove the hMTS Injector.
3. Remove the clear, round Button Cover of the injector by twisting the cover one quarter turn and then pulling the cover away from injector. Set the cover aside.
4. Place injector on the selected injection site.
5. Press the injector to the skin site with minimal pressure so that the adhesive will adhere to the skin.

6. Hold 10 seconds to promote adhesion being careful not to press so hard as to actuate the injector.
7. To actuate the injector, place fingers or palm of hand on the spherical dome of the injector. Press down on the spherical dome quickly to actuate the injector. An audible click will be heard as the injector inserts the microneedle array into the skin. The infusion of the fluid begins immediately upon actuation. The injector will be held securely by the adhesive.
8. Remove hand from the injector.
9. The blue Progress Indicator becomes visible in the clear window during infusion and will advance across the window as fluid is delivered into the intradermal space. NOTE: Depending on the fluid viscosity and delivery location, the infusion may last from 1 to 10 minutes. Patients will be instructed to remain as still as possible.
10. The hMTS device injection is complete when the blue indicator has advanced across the full length of the viewing window.
11. After completion, wait at least 1 minute before removing the injector.
12. To remove the injector, peel off from the skin by placing thumb or finger onto the adhesive tabs and then slowly roll the device to the side to peel adhesive off the skin.

NOTE: No post-delivery treatment is required at the injection site.

NOTE: Transient blanching and erythema may be observed at the injection site. It is normal to see a bleb where the fluid resides until it is absorbed.

15.145 Monitoring of patients after treatment with FRα DCs

Patients will be monitored for 30 minutes following injections for acute toxicity. Expected potential toxicity of vaccine injection includes dermatitis, anaphylaxis, allergic reactions such as bronchospasm or generalized urticaria, autoimmune reactions, bone pain, myalgia/arthralgia, reaction at the site of injection and renal dysfunction.

15.15 **Potential Drug Interactions:** There are no known drug interactions.

15.16 **Known Potential Toxicities:**

15.161 Common toxicities associated with dendritic cell vaccines: injection site reactions, including discomfort, rash, erythema induration, bleeding, tenderness to touch, numbness, tingling, and itching; systemic reactions, including skin rash, itching, sweating, muscle aches, joint aches, and fatigue.

15.162 Rare toxicities associated with dendritic cell vaccines: pain or ulceration at injection site, leucopenia, dyspnea, venous thromboembolism, seizures, arrhythmias, infection, renal insufficiency, hepatitis, headaches, abdominal pain, and cough.

15.163 Toxicities associated with folate receptor alpha vaccines: injection site reactions, leucopenia, and fatigue.

15.17 **Drug Procurement:** Investigational product is provided free of charge to study participants by the Mayo Clinic Human Cellular Laboratory.

15.18 **Nursing guidelines**:

15.181 Patients should be monitored for 30 minutes after injection for acute toxicity.

15.182 Anaphylaxis is a possibility, monitor patients for signs or symptoms of allergic reaction, including hives, swelling, generalized urticaria, or bronchospasm.

15.183 Instruct patients that they may experience mild myalgia/arthralgia and bone pain.

15.184 Warn patients of injection site reactions with may include redness, swelling, and discomfort.

15.185 FRα DC vaccine should only be administered intradermally according to the protocol, by nurses who have been trained in the administration of the vaccine.

16.0 Statistical Considerations and Methodology

16.1 Study design: This is a single-arm pilot study designed to determin the safety and immunogenicity of folate receptor alpha peptide-loaded dendritic cell vaccination in patients with advanced stage epithelial ovarian cancer. The safety lead-in accrual schedule is based on a 3+3 phase I design.

16.11 Accrual and study duration: This pilot study may involve a minimum of 3 patients and maximum of 22 patients. It is expected that the monthly accrual will be 1.5 patients. Thus the accrual portion of the trial will require approximately 15 months. With the 2 planned stops and approximately 2 months required to assess each cohort, the trial will require 19 months to accrue and assess.

16.12 Accrual Schema:

16.121 Safety lead-in:

- Three patients will be accrued and then the study will be temporarily closed to observe for 1 cycle. If there are at least 2 patients with DLTs the study team will decide if further study is desired and a plan will be formulated for the DSMB.
- If there are 0 or 1 patients with DLTs, three more patients will be accrued and the study will be temporarily closed to observe for 1 cycle. If there are at least 2 patients with DLTs (out of the first 6) the study team will decide if further study is desired and a plan will be formulated for the DSMB.

16.122 Remaining accrual: If there are fewer than 2 DLTs (out of the first 6 evaluable patients), the final 16 patients will be accrued.

16.13 Dose limiting toxicities are defined in Section 7.3.

16.2 Analysis plans: All the relevant results pertaining to toxicity, timed endpoints, and laboratory correlates will be examined in an exploratory and hypothesis generating fashion. The small sample size associated with this pilot study restricts the generalization of the results. Any notable statistical result should only be viewed as an impetus for further study in Phase II trials rather than a definitive finding in and of itself.

16.21 Primary Objective: The primary objective of this pilot study is to determine the safety and tolerability of FRαDC vaccination. If the accrual schema (section 16.12) is completed and there are fewer than 5 patients with a DLT, the vaccination treatment will be considered safe in this patient population. The following table presents the probability of having fewer than 5 patients with a DLT under different population rates of DLT.

If the true probability of a single patient having a DLT is…	0.10	0.15	0.20	0.25	0.30
Then the probability that there will be fewer than 4 DLTs (out of 22) is…	0.94	0.77	0.54	0.32	0.16

16.22 Secondary Objectives:

16.221 Time to Disease Recurrence (TDR) is defined as the number of days from study registration until disease recurrence or death. The Kaplan-Meier method will be used to estimate the distribution of TDR.

16.222 Overall Survival (OS) is defined as the number of days from study registration until death due to any cause. The Kaplan-Meier method will be used to estimate the distribution of OS.

16.23 Correlative Objectives: For continuous correlate data, change from baseline measures will be calculated to determine if levels have increased. Simple summary statistics (mean and 95% confidence intervals) will be used to assess. For categorical data (e.g. DTH skin reaction which is positive or negative) the percent of each category will be calculated along with a 95% confidence interval.

16.3 Adverse Event Stopping Rule: The principal investigator and the study statistician will review the study periodically (at least twice a year) to identify accrual, toxicity, and endpoint problems that might be developing. The study statistician will prepare a report containing accrual, adverse event, and efficacy data which will be submitted to the Mayo Clinic Cancer Center Data and Safety Monitoring Board (MCCC DSMB) every 6 months until all patients are off study treatment.

During the first 6 patients, if 2 or more have a DLT (as defined in section 7.3), enrollment will be suspended so that the adverse event data can be examined. A trial recommendation will be formulated and presented to the MCCC DSMB.

At any point in the enrollment process after 6 or more patients have been accrued, if more than 30% of these patients develop a DLT (as defined in section

7.3), enrollment will be suspended so that the adverse event data can be examined. A trial recommendation will be formulated and presented to the MCCC DSMB.

16.4 Inclusion of Women and Minorities:

This study will be available to all eligible patients, regardless of race, gender, or ethnic group. Due to the site of disease, this study will accrue only female patients.

There is no information currently available regarding differential treatment effects in subsets defined by race or ethnicity, and there is no reason to expect such differences exist. Therefore, although the planned analyses will, as always, look for differences in treatment effect based on gender and racial groupings, the samples sizes are not increased in order to provide additional power for such subset analyses.

The geographic area that Mayo Clinic Rochester serves has a very small minority population. It is expected that only 5% of eligible patients are of minority background. Expected sizes of racial by gender subsets are shown in the following table:

Accrual Targets			
**Ethnic Category**	**Sex/Gender**		
	**Females**	**Males**	**Total**
Hispanic or Latino	0	0	0
Not Hispanic or Latino	0	0	0
**Ethnic Category: Total of all subjects***	22	0	22
**Racial Category**			
American Indian or Alaskan Native	0	0	0
Asian	0	0	0
Black or African American	1	0	1
Native Hawaiian or other Pacific Islander	0	0	0
White	21	0	21
**Racial Category: Total of all subjects***	22	0	22

Ethnic Categories:

Hispanic or Latino – a person of Cuban, Mexican, Puerto Rican, South or Central American, or other Spanish culture or origin, regardless of race. The term “Spanish origin” can also be used in addition to “Hispanic or Latino.”

Not Hispanic or Latino

Racial Categories:

American Indian or Alaskan Native – a person having origins in any of the original peoples of North, Central, or South America, and who maintains tribal affiliations or community attachment.

Asian – a person having origins in any of the original peoples of the Far East, Southeast Asia, or the Indian subcontinent including, for example, Cambodia, China, India, Japan, Korea, Malaysia, Pakistan, the Philippine Islands, Thailand, and Vietnam. (Note: Individuals from the Philippine Islands have been recorded as Pacific Islanders in previous data collection strategies.)

Black or African American – a person having origins in any of the black racial groups of Africa. Terms such as “Haitian” or “Negro” can be used in addition to “Black or African American.”

Native Hawaiian or other Pacific Islander – a person having origins in any of the original peoples of Hawaii, Guam, Samoa, or other Pacific Islands.

White – a person having origins in any of the original peoples of Europe, the Middle East, or North Africa.

17.0 Pathology Considerations/Tissue Biospecimens

17.1 Summary table of research tissue specimens to be collected for this protocol

Correlative Study (Section for more information)	Mandatory or Optional	Type of Tissue to Collect	Block, Slides, Core, etc. (# of each to submit)	Within 90 Days of Registration	Process at site? (Yes or No)	Temperature Conditions for Storage /Shipping
Determination of FRα expression in primary tumor	Optional	Formalin Fixed Paraffin Embedded	Six slides, 5 microns thick, on charged slides	Yes	Yes	ambient
Determination of FRα expression in recurrent tumor	Optional	Formalin Fixed Paraffin Embedded	Six slides, 5 microns thick, on charged slides	No	Yes	ambient

17.2 Diagnostic slides from original and /or recurrent tissue

Original diagnostic slides need not be submitted for this study; however the following reports should be submitted

- Pathology reporting form,
- Surgical pathology report
- Operative report.

17.3 Correlative tissue collection

17.31 Paraffin Embedded Tissue (if available)

17.311 Submit 6 slides (5 microns on charged slides)

17.312 Ship all specimens to:
Dr. Kimberly R. Kalli
Charlton 6-118
200 First St. SW
Rochester, MN 55905

17.4 Background and methodology

17.41 FRα staining in tissue specimens.
As a correlative objective, FRα expression in the tumor will be examined as previously described [27]. FRα is expressed in both epithelial (breast, ovarian, uterine, testicular, colon, renal, etc.) and nonepithelial malignancies (myelogenous leukemias and sarcomas). Particular interest in therapeutic targeting of FRα has focused on ovarian cancer because 70-90% of non-mucinous tumors express the protein at high levels [41, 42].

Samples will be examined for FRα expression by assessing slides made from a full paraffin block. Tissues will be stained with FBP343, a monoclonal IgG1

antibody derived by immunization with human FRα purified from the KB nasopharyngeal carcinoma cell line.

Five-micron sections will be cut and placed on positively charged slides. After rehydration, tissues will be subjected to antigen retrieval and blocking of endogenous peroxidases prior to staining with 3.6 µg/ml FBP343 or a non-specific isotype matched antibody as a negative control for 30 min. After washing the slides, signals will be detected using the mouse MACH3 system (Biocare Medical, Walnut Creek, CA). Slides will be counterstained with Modified Schmidt's Hematoxylin and permanently mounted.

Slides will then be archived using digital imaging performed using a Bliss “Virtual Microscopy” microscope and computer system (Bacus Laboratories, Lombard, IL). The staining intensity (strong, moderate, weak, or negative) and proportion of FRα-positive cells among the malignant cells will be scored independently on the digital images by two observers in Dr. Knutson’s and Dr. Kalli’s group who are blinded to all clinical outcome data.

18.0 Records and Data Collection Procedures

18.1 Submission Timetable

18.11 Initial Material(s)

**CRF**	**Active-Monitoring Phase** (Compliance with Test Schedule Section 4.0)
On-Study Form	≤2 weeks after registration
Baseline Adverse Event Form	
Research Blood Submission Form (see Section 14.0)	
Research Tissue Submission Form (see Section 17.0)	
CA 125 Form	
End of Active Treatment/Cancel Notification Form	Submit ≤2 weeks after registration if withdrawal/refusal occurs prior to beginning protocol therapy

18.12 Test Schedule Material(s)

CRF	At each evaluation during treatment	At end of treatment
Evaluation/Treatment Form	X	X
DTH Form	X	X
CA125 Form	X	X
Adverse Event Form	X	X
Active Monitoring Phase Measurement Form	X	X
Research Blood Submission Form	X (see Section 14.0)	X
Research Blood submission Form (at recurrence-Optional	At each occurrence	
Research Tissue Submission Form	X (see Section 17.0)	X
Interval Laboratory Form	X	X
End of Active Treatment/Cancel Notification Form Submit Once Per Patient		X

18.13 Follow-up Material(s)

**CRF**	**Event Monitoring Phase**[1]				
	every 3months until PD	At PD	After PD every. 6 mos.	Death	New Primary
Event Monitoring Form	X	X	X	X	At each occurrence

1. If a patient is still alive 5 years after registration, no further follow-up is required.

19.0 Budget

19.1 Costs charged to patient: conventional clinical care, including clinical evaluation every three months, laboratory tests every three months (hematology group, chemistry group, CA-125), and any required clinical evaluation (clinically indicated CT abdomen/pelvis).

19.2 Tests to be research funded: clinical evaluation when performed more than once within three months, laboratory tests when performed more than once within three months (hematology group, chemistry group), tetanus vaccine treatment, pathology evaluation of tumors for folate receptor alpha, research blood tests, apheresis, treatment with FRα DCs, and DTH testing with unloaded DCs.

19.3 Other budget concerns: none

20.0 References

1. Chobanian N, Dietrich CS, 3rd. Ovarian cancer. Surg Clin North Am 2008;88:285-99, vi.
2. Zhang L, Conejo-Garcia JR, Katsaros D, Gimotty PA, Massobrio M, Regnani G, et al. Intratumoral T cells, recurrence, and survival in epithelial ovarian cancer. N Engl J Med 2003;348:203-13.
3. Curiel TJ, Coukos G, Zou L, Alvarez X, Cheng P, Mottram P, et al. Specific recruitment of regulatory T cells in ovarian carcinoma fosters immune privilege and predicts reduced survival. Nat Med 2004;10:942-9.
4. Wolf D, Wolf AM, Rumpold H, Fiegl H, Zeimet AG, Muller-Holzner E, et al. The expression of the regulatory T cell-specific forkhead box transcription factor FoxP3 is associated with poor prognosis in ovarian cancer. Clin Cancer Res 2005;11:8326-31.
5. Sato E, Olson SH, Ahn J, Bundy B, Nishikawa H, Qian F, et al. Intraepithelial CD8+ tumor-infiltrating lymphocytes and a high CD8+/regulatory T cell ratio are associated with favorable prognosis in ovarian cancer. Proc Natl Acad Sci U S A 2005;102:18538-43.
6. Dong H, Strome SE, Salomao DR, Tamura H, Hirano F, Flies DB, et al. Tumor-associated B7-H1 promotes T-cell apoptosis: a potential mechanism of immune evasion. Nat Med 2002;8:793-800.
7. Curiel TJ, Wei S, Dong H, Alvarez X, Cheng P, Mottram P, et al. Blockade of B7-H1 improves myeloid dendritic cell-mediated antitumor immunity. Nat Med 2003;9:562-7.
8. Wang L, Pino-Lagos K, de Vries VC, Guleria I, Sayegh MH, Noelle RJ. Programmed death 1 ligand signaling regulates the generation of adaptive Foxp3+CD4+ regulatory T cells. Proc Natl Acad Sci U S A 2008;105:9331-6.
9. Sharma MD, Hou DY, Liu Y, Koni PA, Metz R, Chandler P, et al. Indoleamine 2,3-dioxygenase controls conversion of Foxp3+ Tregs to TH17-like cells in tumor-draining lymph nodes. Blood 2009;113:6102-11.
10. Chung DJ, Rossi M, Romano E, Ghith J, Yuan J, Munn DH, et al. Indoleamine 2,3-dioxygenase-expressing mature human monocyte-derived dendritic cells expand potent autologous regulatory T cells. Blood 2009;114:555-63.
11. Hamanishi J, Mandai M, Iwasaki M, Okazaki T, Tanaka Y, Yamaguchi K, et al. Programmed cell death 1 ligand 1 and tumor-infiltrating CD8+ T lymphocytes are prognostic factors of human ovarian cancer. Proc Natl Acad Sci U S A 2007;104:3360-5.
12. Takao M, Okamoto A, Nikaido T, Urashima M, Takakura S, Saito M, et al. Increased synthesis of indoleamine-2,3-dioxygenase protein is positively associated with impaired survival in patients with serous-type, but not with other types of, ovarian cancer. Oncol Rep 2007;17:1333-9.
13. Inaba T, Ino K, Kajiyama H, Yamamoto E, Shibata K, Nawa A, et al. Role of the immunosuppressive enzyme indoleamine 2,3-dioxygenase in the progression of ovarian carcinoma. Gynecol Oncol 2009;115:185-92.

14. Kryczek I, Banerjee M, Cheng P, Vatan L, Szeliga W, Wei S, et al. Phenotype, distribution, generation, and functional and clinical relevance of Th17 cells in the human tumor environments. Blood 2009;114:1141-9.
15. Bettelli E, Oukka M, Kuchroo VK. T(H)-17 cells in the circle of immunity and autoimmunity. Nat Immunol 2007;8:345-50.
16. Kryczek I, Wei S, Zou L, Altuwaijri S, Szeliga W, Kolls J, et al. Cutting edge: Th17 and regulatory T cell dynamics and the regulation by IL-2 in the tumor microenvironment. J Immunol 2007;178:6730-3.
17. Munn DH. Th17 cells in ovarian cancer. Blood 2009;114:1134-5.
18. Schlienger K, Chu CS, Woo EY, Rivers PM, Toll AJ, Hudson B, et al. TRANCE- and CD40 ligand-matured dendritic cells reveal MHC class I-restricted T cells specific for autologous tumor in late-stage ovarian cancer patients. Clin Cancer Res 2003;9:1517-27.
19. Santin AD, Bellone S, Ravaggi A, Roman JJ, Pecorelli S, Parham GP, et al. Induction of tumour-specific CD8(+) cytotoxic T lymphocytes by tumour lysate-pulsed autologous dendritic cells in patients with uterine serous papillary cancer. Br J Cancer 2002;86:151-7.
20. Santin AD, Hermonat PL, Ravaggi A, Bellone S, Pecorelli S, Cannon MJ, et al. In vitro induction of tumor-specific human lymphocyte antigen class I-restricted CD8 cytotoxic T lymphocytes by ovarian tumor antigen-pulsed autologous dendritic cells from patients with advanced ovarian cancer. Am J Obstet Gynecol 2000;183:601-9.
21. Zhao X, Wei YQ, Peng ZL. Induction of T cell responses against autologous ovarian tumors with whole tumor cell lysate-pulsed dendritic cells. Immunol Invest 2001;30:33-45.
22. Gong J, Nikrui N, Chen D, Koido S, Wu Z, Tanaka Y, et al. Fusions of human ovarian carcinoma cells with autologous or allogeneic dendritic cells induce antitumor immunity. J Immunol 2000;165:1705-11.
23. Hernando JJ, Park TW, Kubler K, Offergeld R, Schlebusch H, Bauknecht T. Vaccination with autologous tumour antigen-pulsed dendritic cells in advanced gynaecological malignancies: clinical and immunological evaluation of a phase I trial. Cancer Immunol Immunother 2002;51:45-52.
24. Brossart P, Wirths S, Stuhler G, Reichardt VL, Kanz L, Brugger W. Induction of cytotoxic T-lymphocyte responses in vivo after vaccinations with peptide-pulsed dendritic cells. Blood 2000;96:3102-8.
25. Hernando JJ, Park TW, Fischer HP, Zivanovic O, Braun M, Polcher M, et al. Vaccination with dendritic cells transfected with mRNA-encoded folate-receptor-alpha for relapsed metastatic ovarian cancer. Lancet Oncol 2007;8:451-4.
26. Hartmann LC, Keeney GL, Lingle WL, Christianson TJ, Varghese B, Hillman D, et al. Folate receptor overexpression is associated with poor outcome in breast cancer. Int J Cancer 2007;121:938-42.
27. Kalli KR, Oberg AL, Keeney GL, Christianson TJ, Low PS, Knutson KL, et al. Folate receptor alpha as a tumor target in epithelial ovarian cancer. Gynecol Oncol 2008;108:619-26.
28. Knutson KL, Krco CJ, Erskine CL, Goodman K, Kelemen LE, Wettstein PJ, et al. T-cell immunity to the folate receptor alpha is prevalent in women with breast or ovarian cancer. J Clin Oncol 2006;24:4254-61.
29. Jackson AM, Mulcahy LA, Zhu XW, O'Donnell D, Patel PM. Tumour-mediated disruption of dendritic cell function: inhibiting the MEK1/2-p44/42 axis restores IL-12 production and Th1-generation. Int J Cancer 2008;123:623-32.
30. Xie J, Qian J, Yang J, Wang S, Freeman ME, 3rd, Yi Q. Critical roles of Raf/MEK/ERK and PI3K/AKT signaling and inactivation of p38 MAP kinase in the differentiation and survival of monocyte-derived immature dendritic cells. Exp Hematol 2005;33:564-72.
31. Wang S, Hong S, Yang J, Qian J, Zhang X, Shpall E, et al. Optimizing immunotherapy in multiple myeloma: Restoring the function of patients' monocyte-derived dendritic cells by inhibiting p38 or activating MEK/ERK MAPK and neutralizing interleukin-6 in progenitor cells. Blood 2006;108:4071-7.

32. Jarnicki AG, Conroy H, Brereton C, Donnelly G, Toomey D, Walsh K, et al. Attenuating regulatory T cell induction by TLR agonists through inhibition of p38 MAPK signaling in dendritic cells enhances their efficacy as vaccine adjuvants and cancer immunotherapeutics. J Immunol 2008;180:3797-806.
33. Brereton CF, Sutton CE, Lalor SJ, Lavelle EC, Mills KH. Inhibition of ERK MAPK suppresses IL-23- and IL-1-driven IL-17 production and attenuates autoimmune disease. J Immunol 2009;183:1715-23.
34. Cannon MJ, Goyne HE, Stone PJB. MacDonald LJ, James LE, Cobos E, Chiriva-Internati M. Modulation of p38 MAPK signaling enhances dendritic cell activation of human $CD4^+$ Th17 responses to ovarian tumor antigen. Cancer Immunol Immunother 2013;62:839-49.
35. Paulos CM, Carpenito C, Plesa G, Suhoski MM, Varela-Rohena A, Golovina TN, et al. The inducible costimulator (ICOS) is critical for the development of human T(H)17 cells. Sci Transl Med 2010;2:55ra78.
36. Favre D, Mold J, Hunt PW, Kanwar B, Loke P, Seu L, et al. Tryptophan catabolism by indoleamine 2,3-dioxygenase 1 alters the balance of TH17 to regulatory T cells in HIV disease. Sci Transl Med 2010;2:32ra6.
37. Santin AD, Bellone S, Palmieri M, Zanolini A, Ravaggi A, Siegel ER, et al. Human papillomavirus type 16 and 18 E7-pulsed dendritic cell vaccination of stage IB or IIA cervical cancer patients: a phase I escalating-dose trial. J Virol 2008;82:1968-79.
38. Murugaiyan G, Saha B. Protumor vs antitumor functions of IL-17. J Immunol 2009;183:4169-75.
39. Wilke CM, Kryczek I, Wei S, Zhao E, Wu K, Wang G, Zou W. Th17 cells in cancer: help or hindrance? Carcinogenesis 2011;32:643-9
40. Knutson KL, Schiffman K, Disis ML. Immunization with a HER-2/neu helper peptide vaccine generates HER-2/neu CD8 T-cell immunity in cancer patients. J Clin Invest 2001;107:477-84.
41. Toffoli G, Cernigoi C, Russo A, gallo A, Bagnoli M, Boiocchi M. Overexpression of folate binding protein in ovarian cancers. Int J Cancer 1997;74:193-8.
42. Parker N, Turk MJ, Westrick E, Lewis JD, Low PS, Leamon CP. Folate receptor expression in carcinomas and normal tissues determined by quantitative radioligand binding assay. Anal Biochem 2005;338:284-93.

Appendix I ECOG PERFORMANCE STATUS

ECOG PERFORMANCE STATUS*	
**Grade**	**ECOG**
0	Fully active, able to carry on all pre-disease performance without restriction
1	Restricted in physically strenuous activity but ambulatory and able to carry out work of a light or sedentary nature, e.g., light house work, office work
2	Ambulatory and capable of all selfcare but unable to carry out any work activities. Up and about more than 50% of waking hours
3	Capable of only limited selfcare, confined to bed or chair more than 50% of waking hours.
4	Completely disabled. Cannot carry on any selfcare. Totally confined to bed or chair.
5	Dead

*As published in Am. J. Clin. Oncol.:
Oken, M.M., Creech, R.H., Tormey, D.C., Horton, J., Davis, T.E., McFadden, E.T., Carbone, P.P.: Toxicity And Response Criteria Of The Eastern Cooperative Oncology Group. Am J Clin Oncol 5:649-655, 1982.

The ECOG Performance Status is in the public domain therefore available for public use. To duplicate the scale, please cite the reference above and credit the Eastern Cooperative Oncology Group, Robert Comis M.D., Group Chair.

From http://www.ecog.org/general/perf_stat html

Appendix II INJECTION SITE RECORD

Protocol #: ________________ Patient #: _________________ Patient Initials: ___ ___ ___
F M L

Please indicate on the diagrams below the site of vaccine injection and the site of control DC injection (if applicable). Write “F” for FRαDCs or “U” for unloaded DCs (if applicable).

Date of determination: __ __ / __ __ / __ __
M D Y

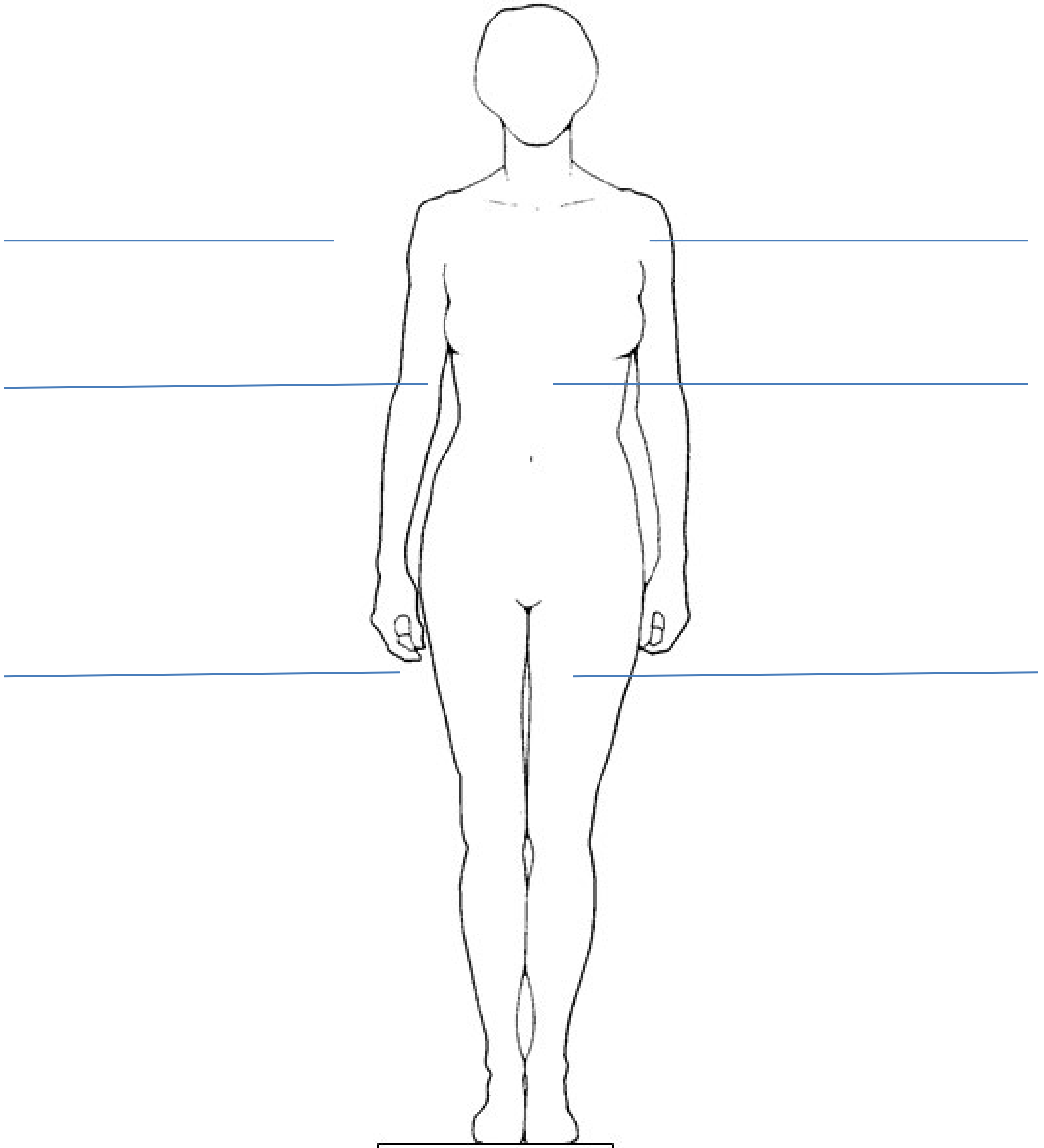

Anterior

Signature_____________________________________ Date_____________________________

Appendix III Clinical Evaluation for Autoimmunity

MC1361 Clinical Evaluation for Autoimmunity and Use of Immune-modulating Drugs
(Evaluator to document examination in clinical note)

**Exam component**	**Status (circle one)**	
Cranial nerves II-XII	Normal	Abnormal*
Cerebellar function	Normal	Abnormal*
Neuromuscular function	Normal	Abnormal*
Joint exam	Normal	Abnormal*
Skin exam	Normal	Abnormal*

*Abnormalities must be documented in the medical record and attributed in the Clinical Document Management (CDM) report.

MC1361 Use of immune-modulating doses of steroids or NSAIDs
(circle answer)

In the past four weeks, has the patient used steroids in excess of 5 mg oral prednisone (or equipotent dose of other steroid) daily (when averaged over the four week period)?

No Yes†

In the past four weeks, has the patient used non-steroidal anti-inflammatory drugs (NSAIDs) in excess of 400 mg oral ibuprofen, 250 mg oral naproxen, or 325 mg oral aspirin (or equipotent dose of other NSAID) daily (when averaged over the four week period)?

No Yes†

† If immune-modulating doses of steroids or NSAIDs were used, this must be documented in the clinical note, along with the reason for use.

Signature______________________________ Date____________________

Appendix IV Patient Measurement of DTH Reactions

Protocol #: ________________ Patient #: _________________ Patient Initials: ___ ___ ___
F M L

Date of determination: __ __ / __ __ / __ __
M D Y

Indicate the extent of redness:
1_____No redness
2_____Distinct red spots
3_____Confluent ring of redness (_____ mm)
4_____Solid red circle (_____ mm)

Indicate the extent of redness:
1_____No redness
2_____Distinct red spots
3_____Confluent ring of redness (_____ mm)
4_____Solid red circle (_____ mm)

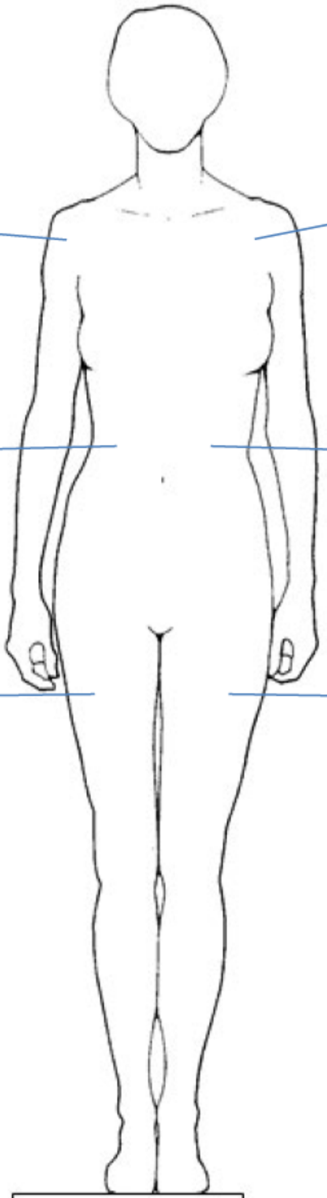

Indicate the extent of redness:
1_____No redness
2_____Distinct red spots
3_____Confluent ring of redness (_____ mm)
4_____Solid red circle (_____ mm)

Indicate the extent of redness:
1_____No redness
2_____Distinct red spots
3_____Confluent ring of redness (_____ mm)
4_____Solid red circle (_____ mm)

Indicate the extent of redness:
1_____No redness
2_____Distinct red spots
3_____Confluent ring of redness (_____ mm)
4_____Solid red circle (_____ mm)

Indicate the extent of redness:
1_____No redness
2_____Distinct red spots
3_____Confluent ring of redness (_____ mm)
4_____Solid red circle (_____ mm)

Anterior

Signature____________________________________ Date_____________________

Appendix V Instruction Page for Patient Measurement of (Delayed-Type Hypersensitivity) DTH Skin Reactions

As part of the clinical trial, you are asked to record measurements of any skin reactions that occur after you receive each dose of vaccine. Please record measurements for each injection you receive. For Cycles 1, 5, and 12, you will have 2 sites to measure. For all other cycles, you will have 1 site to measure. Please circle the area(s) that was (were) injected if this has not already been done. For each site, you will need to determine the extent of redness 2-3 days after vaccine treatment. Please record measurements in millimeters (mm).
If you cannot see any area of redness, simply put a checkmark on the line marked "1_No redness".
If you see red spots where the needles on the device, but they do not merge together, put a checkmark on the line marked "2_Distinct red spots".
If the spots have merged together to form a ring of redness, put a checkmark on the line marked "3_Confluent ring of redness", and indicate the thickness of the ring in mm.
If there is redness on all the skin within the injection ring, put a checkmark on the line marked "4_Solid red circle", and indicate the diameter of the circle.
Please refer to the examples below.

1_____No redness

2_____Distinct red spots

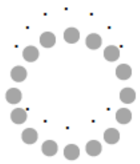

3_____Confluent ring of redness (4 mm)

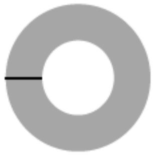

4_____Solid red circle (20 mm)

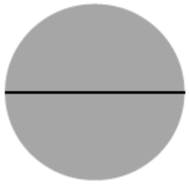

Note: The measurements must be done 2-3 days after the needle stick. Please use the below ruler as a guide when estimating areas of redness.

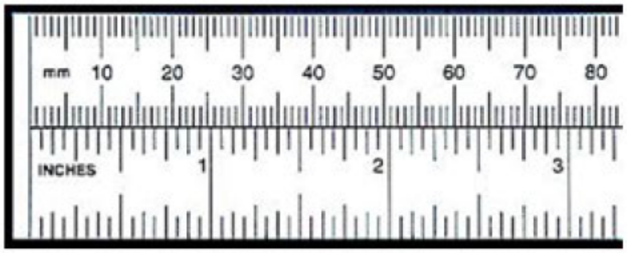

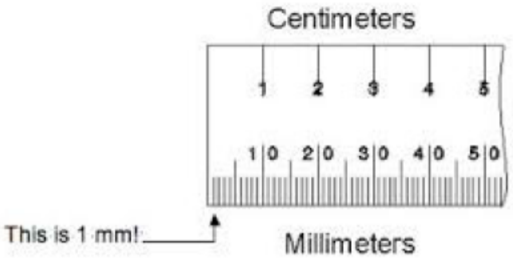

Supplement: Supplementary file 1 — Supplementary Information [file 41467_2020_18962_MOESM1_ESM.pdf]
